# Naoxintong Capsule Inhibits the Development of Cardiovascular Pathological Changes in Bama Minipig Through Improving Gut Microbiota

**DOI:** 10.3389/fphar.2019.01128

**Published:** 2019-10-03

**Authors:** Wei-jian Zhang, Wei-wei Su, Pei-bo Li, Hong-yu Rao, Qing-wei Lin, Xuan Zeng, Tao-bin Chen, Zeng-hao Yan, Hong Liu, Hong-liang Yao

**Affiliations:** ^1^Guangdong Engineering & Technology Research Center for Quality and Efficacy Reevaluation of Post-market Traditional Chinese Medicine, Guangdong Key Laboratory of Plant Resources, State Key Laboratory of Biocontrol, School of Life Sciences, Sun Yat-sen University, Guangzhou, China; ^2^Key Laboratory of Traditional Chinese Medicine Quality Standards, Guangxi Institute of Traditional Medical and Pharmaceutical Sciences, Nanning, China; ^3^Guangdong Key Laboratory of Animal Conservation and Resource Utilization, Guangdong Public Laboratory of Wild Animal Conservation and Utilization, Drug Synthesis and Evaluation Center, Guangdong Institute of Applied Biological Resources, Guangzhou, China

**Keywords:** Naoxintong capsule (NXT), Bama minipig, cardiovascular diseases, blood lipid metabolism, gut microbiota

## Abstract

Naoxintong capsule (NXT), a Chinese medicine, has performed excellent effects on the prevention and treatment against cardiovascular diseases. NXT is a fine powder mixture without any herb extraction, and there must be lots of ingredients hard to be absorbed. However, little is known about the correlation between the NXT’s cardioprotective effects and gut microbiota. Herein, we report the effect of NXT on the development of cardiovascular diseases and clarify the correlation between NXT’s cardioprotective effects and gut microbiota. In the current study, minipigs were selected and fed with high-fat diet and NXT daily for successive 8 months. During the process, up to 18 biomedical parameters were monthly determined to observe the dynamic changes after NXT treatment. At the end of experimental process, pathological examinations of heart, coronary artery, carotid artery, thoracic aorta, and abdominal aorta were conducted by HE staining and 16SrDNA sequencing, and analyzing of gut microbiota were conducted. Our results showed that NXT’s effects against cardiovascular diseases were through regulating blood lipid profiles, inhibiting vascular inflammation, enhancing antioxidant capacity, and alleviating myocardial injury, without damages on liver and kidney particularly. Concurrently, we also found that long-term administration of NXT increased the diversity of gut microbiota, influenced the microbiome structure and composition stably, and revered the increase of the ratio of the *Firmicutes* to *Bacteroidetes* (F/B ratio) in relative abundance. Specifically, our results revealed some key bacterium of *Caproiciproducens (enhanced)*, *Sutterella (enhanced)*, *Erysipelotrichaceae (enhanced)*, and *Romboutsia (decreased)* that were closely involved in NXT’s effects. Taken together, our study demonstrates that NXT can inhibit the development of cardiovascular diseases by ameliorating high-fat diet–induced metabolic disorders and partly through improving gut microbiota.

**Graphical Abstract:**
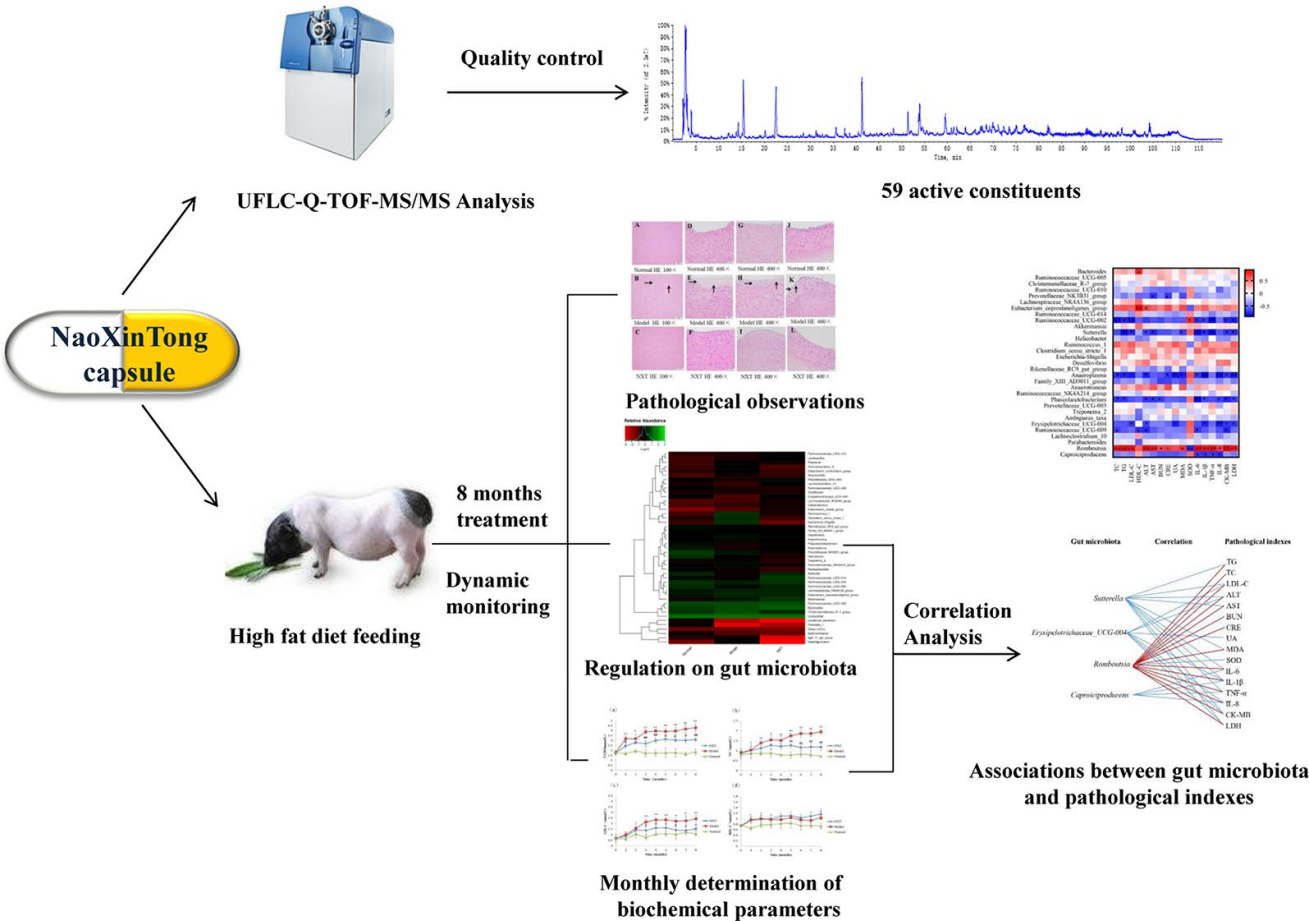
The graphic abstract of the study.

## Introduction

Atherosclerotic cardiovascular diseases are the leading cause of morbidity and mortality worldwide. Dyslipidemia is one of the most common risk factors leading to cardiovascular diseases (Besli et al., 2017). High-fat diet is considered to be the indispensable factor to induce lipid metabolism disorder and result in atherosclerotic cardiovascular disease. Studies *in vivo* and *in vitro* have demonstrated that high-fat feeding could cause inflammation (Jakobsdottir et al., 2013), induce oxidative stress (Paula et al., 2016), generate endothelial dysfunctions (Knight et al., 2008), as well as exacerbate hepatic and renal injuries (Michal et al., 2004; Pinhal et al., 2013). Such high-fat diet–induced metabolic disorders are closely associated with the generation and development of atherosclerotic cardiovascular disease.

Minipigs, smaller than domestic swine, are becoming increasingly attractive experimental animal for research. Minipigs have been proven to be recommended model for hyperlipidemia, atherosclerosis, and cardiovascular studies because the morphology and function of their cardiovascular system closely resemble that of humans. More importantly, minipigs have lipoprotein profiles and metabolism similar to humans, and they develop atherosclerosis with increased age spontaneously (Smith et al., 1990; Bergen and Mersmann, 2005; Casellas et al., 2013). And giving minipigs high-fat diet feeding can induce hyperlipidemia and atherosclerosis similar to that of humans (Shoumin et al., 2004; Miyoshi et al., 2010).

In recent years, numerous studies have demonstrated that gut microbiota played a key role in the maintenance of human health, and many inflammatory and metabolic diseases are related to the imbalance of intestinal microecology (Nadja et al., 2010; Vrieze et al., 2010). Long-term high-fat diet intervention can change the intestinal microecology, and the imbalance of gut microbiota could cause the disorder of lipid metabolism, thus leading to cardiovascular disease (Zhang et al., 2010; Murphy et al., 2015).

NXT has been widely used in the prevention and treatment of coronary heart disease, stroke, and other vascular diseases since it was listed in 1993. It is composed of 16 kinds of traditional Chinese medicines, including *Astragalus membranaceus*, *Salvia miltiorrhiza*, *Ligusticum*, *Radix paeoniae rubra*, *Szechwan lovage Rhizome*, *Semen persicae*, *Carthamus tinctorius* L., *Frankincense*, *myrrh*, *Spatholobus suberectus*, *Achyranthes root*, *Cassia Twig*, *Mulberry Twig*, *Buthus martensii*, *Hirudo nipponica*, and *Pheretima aspergillum*. Numerous studies *in vivo* have demonstrated that NXT had protective effects in vascular diseases through different actions such as lowering glucose and lipid, improving ischemia reperfusion injury, stabilizing vulnerable plaques, etc. (Wang et al., 2017; Yang et al., 2017; Yang et al., 2018). Ma X H et al. analyzed the absorbable components of NXT and used the network pharmacology method to discuss the mechanism of NXT in the treatment of heart diseases, and their result showed that NXT significant regulated 123 targets and related pathways (Ma et al., 2016). Recently, Yang X X et al. summarized the cardioprotective properties and the involved mechanisms of NXT, and the review shows that multiple protective effects of NXT on cardiovascular diseases can be correlated to the actions of NXT on inflammation, oxidative stress, and lipid/glucose metabolism (Han et al., 2019).

Different from general Chinese traditional medicine, NXT is a fine powder mixture containing herbs without any extraction. Therefore, there are some components hard to be absorbed, such as a large number of dietary fibers. Accordingly, this study was designed on the hypothesis that NXT’s effects against the cardiovascular diseases are partly through ameliorating high-fat diet–induced metabolic disorders and partly through improving gut microbiota. Consequently, we attempted to perform long-term dynamic observation of NXT against the development of cardiovascular pathological changes, including the effects of NXT on blood lipid profiles, inflammation, oxidative stress, liver and kidney functions, myocardial enzyme, as well as the change of gut microbiota in Bama minipigs, along with long-term high-fat diet feeding and medication treatment.

## Materials and Methods

### Drugs and Reagents

NXT (Med-drug permit no. Z20025001) was kindly provided by Buchang Pharmaceutical Co., Ltd. NXT is composed of 16 herbs including *A. membranaceus* (165 g/kg, g weight of herb/kg weight of NXT powder), *Radix paeoniae rubra* (67.5 g/kg), *Salvia miltiorrhiza* (67.5 g/kg), *Ligusticum* (67.5 g/kg), *Szechwan lovage Rhizome* (67.5 g/kg), *Semen persicae* (67.5 g/kg), *Achyranthes root* (67.5 g/kg), *Mulberry twig* (67.5 g/kg), *P. aspergillum* (67.5 g/kg), *Hirudo nipponica* (67.5 g/kg), *Spatholobus suberectus* (50 g/kg), *Cassia Twig* (50 g/kg), *Carthamus tinctorius* L. (32.5 g/kg), *Frankincense* (32.5 g/kg), *myrrh* (32.5 g/kg), and *Buthus martensii* (32.5 g/kg). All the above herbs, in the radios of 66:27:27:27:27:27:27:27:27:27:20:20:13:13:13:13 (dry weight), are crushed into fine powder, screened through mesh size of 80, and mixed homogeneously, without any extraction (Chinese Pharmacopoeia Commission, 2015). Assay kits for total cholesterol (TC), triglyceride (TG), high-density lipoprotein cholesterol (HDL-C), low-density lipoprotein (LDL-C), alanine transaminase (ALT), aspartate transaminase (AST), blood urea nitrogen (BUN), creatinine (CRE), uric acid (UA), methane dicarboxylic aldehyde (MDA), superoxide dismutase (SOD), lactic dehydrogenase (LDH), and creatine kinase-MB (CK-MB) were purchased from Nanjing Jiancheng Bioengineering Institute Co., Ltd, China. ELISA kits for interleukin-1β (IL-1β), interleukin-8 (IL-8), interleukin-6 (IL-6), and tumor necrosis factor-α (TNF-α) were purchased from cloud-clone Corp., USA. Eosin, hematoxylin, and oil red O were purchased from Shanghai Aladdin Bio-Chem Technology Co., Ltd.; cholesterol was purchased from Guangzhou NOCIKAR Biotechnology Co., Ltd.; butter was purchased from Jieshou Yutu Edible Oil Co., Ltd.; peanut oil was purchased from Shandong Luhua Co., Ltd.; bile salt was purchased from China National Pharmaceutical Group Corporation Chemical Reagent Co., Ltd.; and basic feed were all provided by Dongguan Pearl Lab Animal Sci & Tech Co., Ltd.

### Instruments

BS-3000A electronic analytical balance (Shanghai Yousheng Weighing Apparatus Co., Ltd.), DS-261 Automatic Biochemical Analyzer (Jiangsu SINNOWA Co., Ltd.), KDC-2046 low-speed refrigerated centrifuge (USTC Zonkia Scientific Instruments Co., Ltd.), Haier DW-86L628 Ultra-Low Temperature Refrigerator (Qingdao Haier), OLYMPUS^®^ Biological Microscope (Beijing Cnrico Technology Co., Ltd.), hybrid triple quadrupole time-of-flight mass spectrometer (AB SCIEX Triple TOF™ 5600 plus).

### UFLC-Q-TOF-MS/MS Analysis of NXT

The capsule of NXT was completely removed, and the powder was weighed 1g precisely. The powder was treated by ultrasonic wave in 50ml of 75% methanol for 30 min. The supernatant was filtrated and then injected into ultra-fast liquid chromatography/quadrupole-time-of-flight tandem mass spectrometry system for analysis (UFLC-Q-TOF-MS/MS). The column was a Ultimate XB-C18 (4.6×250mm, 5μm, Welch), which is maintained at 30°C. The mobile phases were composed of acetonitrile (A) and water with 0.1% formic acid (B) using a multi-step linear gradient elution of 10% A at 0–5min, 10–25% A at 5–35min, 25–60% A at 35–65min, 60–67% A at 65–70min, 67% A at 70–80min, 67–90% A at 80–95min, 90% A at 95–105min, 90–10% A at 105–110min, and 10% A at 110–120min with the flow rate kept at 1.0 ml/min. Other MS parameters were adopted same as published work (Zeng et al., 2018). The sample volume injected was set at 20μl. All the acquisition and analysis of data were controlled by the PeakView Software TM V. 1.1 (AB SCIEX, Foster City, CA).

### Ethical Statement and Animals

Nine male Bama minipigs aged 6 months, weighing 13∼18 kg, were provided by Dongguan Pearl Lab Animal Sci & Tech Co., Ltd. (Certification No. SYXK (Yue) 2017-0123). The animals were maintained at the Animal Center of Dongguan Pearl Lab Animal Sci & Tech Co., Ltd. Animals were individually housed in pens under controlled conditions (temperature was maintained at 18∼26°C, and the relative humidity at 40∼70%, with a 12-h light/dark cycle). All the experimental procedures were carried out in accordance with the National Institutes of Health guide for the care and use of laboratory animals and were approved by Animal Care and Use Committee of School of Life Sciences of Sun Yat-sen University. During the experiment, appropriate procedures were taken to minimize the harm to the animals.

### Modeling and Grouping Administration

Bama minipigs were randomly divided into three groups (3/group) and received the following treatment for 8 months: normal group, normal diet; model group, high-fat diet; and NXT group, high-fat diet containing NXT powder (110 mg/kg/day). Food, 3% of body weight/day, was supplied twice a day, and body weights were measured once a month. Normal diet was composed of corn 48%, wheat bran 20%, soybean meal 15%, rice bran 12%, and fish powder 5%. High-fat diet was formed from a mixture of 3% cholesterol, 10% butter, 6% peanut oil, and 0.5% bile salt with normal diet. In order to avoid stress reaction in animals fed with high-fat diet, the proportion of fat was gradually increased in the first 10 days. After the animals get used to the high-fat diet, we started the experiment.

### Determination of Biochemical Parameters

During the experiment, blood samples for measuring biochemical parameters of blood lipid metabolism, hepatic and renal functions, oxidative stress, inflammatory, and myocardial enzyme were collected every month. The animals were anesthetized by intramuscular injection of pentobarbital sodium (30 mg/kg) fasted overnight, and blood was drawn from precaval vein. Ten milliliters of non-anticoagulant blood was standing for 30min at room temperature, and serum was separated from blood by centrifugation for 15min at 3,000 rpm. Six hundred microliters of serum was put into DS-261 automatic biochemical analyzer to detect the TC, TG, HDL-C, LDL-C, ALT, AST, BUN, CRE, CK-MB, and LDH. Four hundred microliters of serum was used to detect the IL-6, IL-8, IL-1β, and TNF-α through the ELISA kits. Fifty microliters was used to detect the SOD and MDA through the colorimetric-assay kits. All the detection operations were conducted in strict according to the corresponding kit instructions.

### Collection of Tissues and Pathological Observation

At the end of the experiment, animals were sacrificed by phlebotomy under anesthesia with intramuscular Injection of pentobarbital sodium (30 mg/kg). The tissues including fresh heart, coronary artery, carotid artery, and abdominal aorta were removed and fixed in 4% paraformaldehyde. And these tissues were made into routine paraffin sections and stained routinely with hematoxylin and eosin (HE) and then examined histopathologically.

### Extraction and Detection of Fecal Genomic DNA and 16SrDNA Gene Sequencing

Fresh fecal samples were collected from each minipig at the end of the experiment, and the fecal DNA was extracted using PowerSoil DNA Extraction Kit (MO BIO Laboratories, Inc., Carlsbad, CA, USA). The quality and concentration of extracted DNA was detected using a Nanodrop ND-1000 Spectrophotometer (Thermo Electron Corporation, USA). PCR amplification of 16SrDNA sequences was performed using primer sets specific for v3–v4 variable regions. Final PCR products were purified from unincorporated nucleotides and primers using the QIAquick PCR Purification Kit (Qiagen, Valencia, USA). Purified samples were normalized to equal DNA concentration and sequenced using the Illumina MiSeq sequencer (Illumina, USA).

### Statistical Analysis

16SrDNA gene sequence data were analyzed using the Quantitative Insights into Microbial Ecology (QIIME) 1.8.0 software (Caporaso et al., 2010). The operational taxonomic units (OTUs) were selected by clustering sequences with a similarity of >97% using USEARCH 7.0.1090 software (Edgar, 2013), and subsequently, chimera detection was performed using the UCHIME method (Edgar et al., 2011). OTUs were annotated with taxonomic information based on RDP classifier version 2.2 algorithm using the Greengenes database.

Alpha diversity indexes were calculated by Mothur v1.31.2 software, and the difference of alpha diversity was evaluated by Kruskal–Wallis test. Beta diversity was measured by calculating the Bray–Curtis. Nonmetric multidimensional scaling (NMDS) was applied on the distance metrics to generate two-dimensional plots in QIIME v1.80. The significant changed bacteria were tested by Metastats (http://metastats.cbcb.umd.edu/). P-value was corrected by p.adjust in the R (v3.0.3) with the method of Benjamini–Hochberg. Correlation between pharmacological indexes and 16SrDNA sequence data are presented in the form of a heatmap diagram based on Spearman’s correlation coefficient. At the genus level, the correlation coefficient of R value was color-mapped onto the gut microbiota, showing how correlated each gut microbiota was with the pharmacological indexes. R values are presented in different colors, and the color card as the color partition of different r values (p < 0.05^*^, p < 0.01^**^).

Apart from the gut microbiota analysis, all the other data were statistically analyzed by Student’s t-test using SPSS software (version: 21.0). The data were expressed as mean ± SD, and a P value less than 0.05 or 0.01 was considered to be statistically significant.

## Results

### Identification of Major Components in NXT

This study employed UFLC-Q-TOF-MS/MS detection to investigate the ingredients of NXT for the quality control. As a result, a total of 59 compounds were identified in NXT based on high-accuracy protonated precursors and multi-stage mass spectrometry according to the reported literature (Wang et al., 2015; Ma et al., 2016) and online databases such as ChemSpider (www.chemspider.com) and the Mass Bank (www.massbank.jp) (details in **Table 1**). These compounds mainly included flavones, flavone glycosides, phenanthraquinones, and terpenoids. Most of these constituents have been reported to show potentially important therapeutic activities for CVDs (Ma et al., 2016). In addition, to ensure the identity of chemical constituents of NXT, the comparison of chemical constituents in three different batches of NXT was carried out, and the results showed the above ingredients of NXT could stably recur (details in **Figure S1**).

**Table 1:** Identification of the chemical constituents of NXT by UFLC-Q-TOF-MS/MS.

***T*** **_R_** **(min)**	Formula	[M+H]^+^ (error, ppm)	[M−H]^−^ (error, ppm)	Fragment ions in positive (+) ion mode	Fragment ions in negative (−) ion mode	Identification
4.02	C_10_H_13_N_5_O_4_	268.1043 (1)		136.0644[M+H−C_5_H_2_N_5_]^+^		Adenosine
5.03	C_7_H_6_O_5_		169.0151 (5.2)		125.0243[M−H−C_2_H_4_O]^−^	Gallic acid
12.21	C_19_H_28_O_11_		431.1559 (0.1)		269.1029[M−H−C_6_H_10_O_5_]^−^	Benzyl-β-gentiobioside
12.22	C_26_H_32_O_14_		567.1721 (0.2)		405.1124[M−H−C_6_H_10_O_5_]^−^, 243.0652[M−H−2C_6_H_10_O_5_]^−^	Mulberroside A
13.05	C_23_H_28_O_12_		495.1511 (0.5)		333.1036[M−H−C_6_H_10_O_5_]^−^	Oxypaeoniflorin
13.39	C_7_H_6_O_3_		137.0260 (10)		108.0206[M−H−CHO]^−^	Protocatechuic aldehyde
13.91	C_16_H_18_O_9_		353.0883 (1.1)		191.0573[M−H−C_6_H_10_O_5_]^−^	Chlorogenic acid
14.27	C_15_H_14_O_6_	291.0866 (0.9)	289.0721 (1.3)	165.0565[M+H−C_6_H_6_O_3_]^+^, 139.0401[M+H−C_8_H_6_O_3_]^+^, 123.0477[M+H−C_8_H_7_O_4_]^+^	245.0859[M−H−CO_2_]^−^, 203.0734[M−H−CO_2_−C_2_H_2_O]^−^, 161.0602[M−H−CO_2_−2C_2_H_2_O]^−^, 151.0422[M−H−C_6_H_6_O_2_−CO]^−^	Catechin
14.34	C_27_H_32_O_16_		611.1622 (0.8)		491.1219[M−H−C_4_H_8_O_4_]^−^, 473.0862[M−H−C_4_H_8_O_4_−H_2_O]^−^, 403.1008[M−H−C_7_H_10_O_6_−H_2_O]^−^, 325.0727[M−H−C_5_H_8_O_5_−H_2_O]^−^, 283.0646[M−H−C_4_H_8_O_4_−C_7_H_10_O_6_−H_2_O]^−^	Hydroxysafflor yellow A
14.44	C_8_H_8_O_5_		183.0308 (3.3)		124.0148[M−H−C_2_H_5_O_2_]^−^	Methyl gallate
15.43	C_20_H_27_NO_11_		456.1512 (0.1)		323.0964[M−H−C_8_H_7_NO]^−^	Amygdalin
19.34	C_15_H_14_O_6_	291.0866 (0.8)	289.0723 (1.8)	165.0565[M+H−C_6_H_6_O_3_]^+^, 139.0401[M+H−C_8_H_6_O_3_]^+^, 123.0477[M+H−C_8_H_7_O_4_]^+^	245.0832[M−H−CO_2_]^−^, 205.0552[M−H−2C_2_H_2_O]^−^, 203.0735[M−H−CO_2_−C_2_H_2_O]^−^, 151.0443[M−H−C_6_H_6_O_2_−CO]^−^, 125.0230[M−H−C_9_H_8_O_3_]^−^	Epicatechin
20.13	C_23_H_28_O_11_	481.1704 (0)	479.1560 (0.3)	197.0807[M+H−C_6_H_10_O_5_−C_7_H_5_O_2_]^+^, 179.0696[M+H−C_6_H_10_O_5_−C_7_H_5_O_2_−H_2_O]^+^	121.0311[M−H−C_15_H_18_O_10_]^−^	Albiflorin
22.04	C_27_H_30_O_17_	627.1550 (−1)	625.1420 (1)	465.1050[M+H−C_6_H_10_O_5_]^+^, 303.0474[M+H−2C_6_H_10_O_5_]^+^	463.0879[M−H−C_6_H_10_O_5_]^−^, 301.0346[M−H−2C_6_H_10_O_5_]^−^	6-Hydroxy kaempferol-di-O-glucoside
22.54	C_23_H_28_O_11_		479.1560 (0.3)		327.1174[M−H−C_7_H_6_O_2_−CH_2_O]^−^, 165.0577[M−H−C_17_H_14_O_6_]^−^, 121.0298[M−H−C_16_H_22_O_9_]^−^	Paeoniflorin
25.12	C_27_H_30_O_16_	611.1601 (−0.9)	609.1471 (0.8)	465.0915[M+H−C_6_H_10_O_4_]^+^, 303.0479[M+H−C_12_H_20_O_9_]^+^	301.0356[M−H−C_12_H_20_O_9_]^−^, 272.0279[M−H−C_13_H_22_O_10_]^−^	Rutin
26.18	C_21_H_20_O_12_	465.1029 (0.2)	463.0887 (1)	303.0490[M+H−C_6_H_10_O_5_]^+^	301.0365[M−H−C_6_H_10_O_5_]^−^	Isoquercitrin
27.2	C_22_H_22_O_10_	447.1285 (−0.3)		285.0746[M+H−C_6_H_10_O_5_]^+^, 270.0514[M+H−C_6_H_10_O_5_−CH_3_]^+^, 253.0474[M+H−C_6_H_10_O_5_−CH_3_OH]^+^, 225.0520[M+H−C_6_H_10_O_5_−CH_3_OH−CO]^+^, 137.0250[M+H−C_6_H_10_O_5_−C_9_H_8_O_2_]^+^		Calycosin-7-O-β-D-glycoside
27.93	C_10_H_10_O_4_	195.0650 (−0.9)	193.0512 (3)	177.0527[M+H−H_2_O]^+^, 149.0594[M+H−H_2_O−CO]^+^, 145.0283[M+H−H_2_O−CH_3_OH]^+^, 134.0390[M+H−H_2_O−CO−CH_3_]^+^, 117.0380[M+H−H_2_O−CO−CH_3_OH]^+^, 89.0445[M+H−H_2_O−CH_3_OH−2CO]^+^	178.0258[M−H−CH_3_]^−^, 134.0371[M−H−CO_2_]^−^, 89.0411[M−H−CH_3_−CO_2_]^−^	Ferulic acid
28.45	C_27_H_44_O_7_	481.3157 (−0.5)		445.2911[M+H−2H_2_O]^+^, 427.2891[M+H−3H_2_O]^+^, 371.2204[M+H−3H_2_O−CO_2_]^+^		β-Ecdysterone
29.88	C_30_H_32_O_15_		631.1679 (1.6)		613.1554[M−H−H_2_O]^−^, 491.0970[M−H−C_7_H_5_O_2_−H_2_O]^−^, 313.0580[M−H−C_17_H_16_O_5_−H_2_O]^−^	Galloylpaeoniflorin
31.01	C_15_H_12_O_7_		303.0515 (1)		285.0535[M−H−H_2_O]^−^, 125.0231[M−H−C_6_H_4_O_3_−3H_2_O]^−^	Taxifolin
31.46	C_41_H_32_O_26_		939.1140 (2.2)		769.0530[M−H−C_7_H_6_O_5_]^−^	Pentagalloylglucose
32.19	C_15_H_10_O_6_	287.0551 (0.4)		258.0529[M+H−CHO]^+^, 213.0552[M+H−H_2_O−2CO]^+^, 165.0163[M+H−C_7_H_6_O_2_]^+^		Kaempferol
32.19	C_21_H_20_O_11_	449.1074 (−0.9)		287.0532[M+H−C_6_H_10_O_5_]^+^		Kaempferol-3-O-glucoside
32.24	C_27_H_30_O_15_	595.1653 (−0.8)	593.1521 (0.8)	287.0548[M+H−C_12_H_20_O_9_]^+^	285.0410[M−H−C_12_H_20_O_9_]^−^	Kaempferol-3-O-rutinoside
33.37	C_14_H_12_O_4_	245.0810 (0.8)	243.0668 (2.3)	199.0777[M+H−C_2_H_6_O]^+^, 181.0603[M+H−C_2_H_6_O−H_2_O]^+^, 161.0606[M+H−C_4_H_4_O_2_]^+^, 107.0544[M+H−C_7_H_4_O_2_−H_2_O]^+^	225.0572[M−H−H_2_O]^−^, 199.0766[M−H−C_2_H_4_O]^−^, 175.0763[M−H−C_4_H_4_O]^−^, 159.0459[M−H−C_4_H_4_O_2_]^−^	Oxyresveratrol
35.71	C_25_H_24_O_13_	533.1284 (−1)		285.0759[M+H−C_6_H_10_O_5_−C_3_H_2_O_3_]^+^		Calycosin-7-O-β-D-glc-6”-O-malonate
36.01	C_9_H_16_O_4_		187.0989 (4.5)		125.0970[M−H−CO_2_−H_2_O]^−^	Skinorim
37.33	C_9_H_6_O_2_	147.0442 (1)		103.0596[M+H−CO_2_]^+^, 91.0593[M+H−2CO]^+^, 77.0471[M+H−C_3_H_2_O_2_]^+^, 65.0479[M+H−C_4_H_2_O_2_]^+^, 51.0300[M+H−C_5_H_4_O_2_]^+^		Coumarin
37.56	C_18_H_16_O_8_		359.0778 (1.6)		197.0474[M−H−C_9_H_6_O_3_]^−^, 179.0358[M−H−C_9_H_6_O_3_−H_2_O]^−^, 161.0247[M−H−C_9_H_6_O_3_−2H_2_O]^−^, 123.0419[M−H−C_11_H_8_O_6_]^−^	Rosmarinic acid
38.19	C_12_H_14_O_3_	207.1018 (0.9)		189.0890[M+H−H_2_O]^+^, 179.1067[M+H−CO]^+^, 165.0873[M+H−C_2_H_2_O]^+^, 161.0943[M+H−H_2_O−CO]^+^		Senkyunolide F
38.71	C_26_H_22_O_10_		493.1151 (2.2)		295.0623123.0419[M−H−C_9_H_10_O_5_]^−^, 159.0453[M−H−C_9_H_10_O_5_−C_8_H_8_O_2_]^−^, 109.0290[M−H−C_9_H_10_O_5_−C_11_H_6_O_3_]^−^	Salvianolic acid A
40.71	C_22_H_22_O_9_	431.1338 (0.2)		269.0813[M+H−C_6_H_10_O_5_]^+^, 254.0563[M+H−C_6_H_10_O_5_−CH_3_]^+^, 237.0533[M+H−C_6_H_10_O_5_−CH_3_OH]^+^, 213.0902[M+H−C_6_H_10_O_5_−2CO]^+^		Ononin
41.36	C_36_H_30_O_16_	719.1602 (−0.7)	717.1488 (3.2)	521.0991[M+H−C_9_H_10_O_5_]^+^, 341.0605[M+H−C_9_H_10_O_5_−C_9_H_8_O_4_]^+^, 323.0536[M+H−2C_9_H_10_O_5_]^+^	519.0952[M−H−C_9_H_10_O_5_]^−^, 339.0531[M−H−C_9_H_10_O_5_−C_9_H_8_O_4_]^−^, 321.0413[M−H−2C_9_H_10_O_5_]^−^, 295.0630[M−H−C_9_H_10_O_5_−C_9_H_8_O_4_−CO_2_]^−^, 279.0307[M−H−2C_9_H_10_O_5_−C_2_H_2_O]^−^	Salvianolic acid B
45.27	C_16_H_12_O_5_	285.0760 (0.9)	283.0620 (3)	270.0516[M+H−CH_3_]^+^, 253.0470[M+H−CH_3_OH]^+^, 225.0540[M+H−CH_3_OH−CO]^+^, 197.0580[M+H−CH_3_OH−2CO]^+^, 137.0245[M+H−C_9_H_8_O_5_]^+^	268.0385[M−H−CH_3_]^−^, 195.0483[M−H−CH_3_OH−2CO]^−^, 135.0056[M−H−C_9_H_8_O_5_]^−^	Calycosin
46.93	C_17_H_16_O_5_	301.1073 (0.9)	299.0933 (2.7)	167.0714[M+H−C_8_H_6_O_2_]^+^,		(6aR,11aR)-3-Hydroxy-9,10-dimethoxypterocarpan
48.25	C_30_H_32_O_12_		583.1829 (1.4)		165.0501[M−H−C_24_H_18_O_7_]^−^, 121.0278[M−H−C_23_H_26_O_10_]^−^	Benzoylpaeoniflorin
49.21	C_9_H_8_O	133.0648 (0.4)		115.0581[M+H−H_2_O]^+^, 105.0755[M+H−CO]^+^, 91.0609[M+H−C_2_H_2_O]^+^, 77.0468[M+H−C_3_H_4_O]^+^		Cinnamaldehyde
51.46	C_43_H_42_O_22_		909.2125 (3.3)		501.1065[M−H−C_19_H_20_O_10_]^−^, 407.0936[M−H−C_24_H_22_O_12_]^−^	Carthamin
53.9	C_42_H_66_O_14_		793.4401 (2.7)		631.3946[M−H−C_6_H_10_O_5_]^−^	Chikusetsusaponin IVa
54.24	C_47_H_72_O_20_		955.4577 (3.4)		835.4515[M−H−C_4_H_8_O_4_]^−^, 793.4461[M−H−C_6_H_10_O_5_]^−^	Achyranthoside C
54.26	C_47_H_70_O_20_		953.4443 (4.8)		909.4571[M−H−CO_2_]^−^, 851.4411[M−H−C_4_H_8_O_4_]^−^, 793.4462[M−H−C_5_H_4_O_6_]^−^	Achyranthoside A
54.46	C_12_H_14_O_3_		205.0874 (2.1)		161.0962[M−H−CO_2_]^−^	3-Butyl-4-hydroxyphthalide
54.82	C_16_H_12_O_4_	269.0810 (0.8)	267.0666 (1.3)	254.0567[M+H−CH_3_]^+^, 237.0527[M+H−CH_3_OH]^+^, 213.0892[M+H−2CO]^+^	252.0428[M−H−CH_3_]^+^	Formononetin
60.83	C_19_H_18_O_4_	311.1281 (1)		267.1380[M+H−CO_2_]^+^		Hydroxytanshinone IIA
62.89	C_12_H_16_O_2_	193.1225 (0.9)		147.1187[M+H−H_2_O−CO]^+^, 137.0624[M+H−C_4_H_8_]^+^, 105.0745[M+H−H_2_O−CO−C_3_H_6_]^+^, 91.0606[M+H−H_2_O−CO−C_4_H_8_]^+^		Senkyunolide A
67.87	C_12_H_18_O_2_	195.1378 (−0.7)		177.1309[M+H−H_2_O]^+^, 149.1345[M+H−H_2_O−CO]^+^		Neocnidilide
68.04	C_18_H_14_O_3_	279.1018 (0.8)		261.0910[M+H−H_2_O]^+^, 233.0959[M+H−H_2_O−CO]^+^, 205.1012[M+H−H_2_O−2CO]^+^, 190.0776[M+H−H_2_O−2CO−CH_3_]^+^		Dihydrotanshinone I
68.42	C_12_H_14_O_2_	191.1066 (−0.4)		173.0976[M+H−H_2_O]^+^, 163.1125[M+H−C_2_H_4_]^+^, 155.0866[M+H−2H_2_O]^+^, 149.0612[M+H−C_3_H_6_]^+^, 145.1029[M+H−H_2_O−CO]^+^, 135.0462[M+H−C_4_H_8_]^+^		Ligustilide
70.72	C_20_H_18_O_5_	339.1230 (1)		279.1003[M+H−C_2_H_4_O_2_]^+^, 261.0908[M+H−C_2_H_4_O_2_−H_2_O]^+^, 233.0956[M+H−C_2_H_4_O_2_−H_2_O−CO]^+^, 205.0994[M+H−C_2_H_4_O_2_−H_2_O−2CO]^+^, 190.0763[M+H−C_2_H_4_O_2_−H_2_O−2CO−CH_3_]^+^		Methyl tanshinonate
74.36	C_19_H_20_O_3_	297.1492 (2.1)		279.1372[M+H−H_2_O]^+^, 268.1080[M+H−C_2_H_4_]^+^, 251.1422[M+H−H_2_O−CO]^+^, 237.0896[M+H−H_2_O−C_3_H_6_]^+^, 209.0957[M+H−H_2_O−C_5_H_10_]^+^		Cryptotanshinone
74.79	C_18_H_32_O_3_		295.2292 (3.9)		277.2176[M−H−H_2_O]^−^, 195.1400[M−H−C_6_H_12_O]^−^	13-Hydroxy-9,11-hexadecadienoic acid
75.02	C_18_H_12_O_3_	277.0862 (0.8)		249.0908[M+H−CO]^+^, 231.0756[M+H−CO−H_2_O]^+^, 221.0990[M+H−C_3_H_4_O]^+^, 193.1013[M+H−2CO]^+^		Tanshinone I
83.42	C_24_H_28_O_4_	381.2063 (0.2)		191.1069[M+H−C_12_H_14_O_2_]^+^, 173.0970[M+H−C_12_H_14_O_2_−H_2_O]^+^, 149.0623[M+H−C_12_H_14_O_2_−C_3_H_6_]^+^, 135.0470[M+H−C_12_H_14_O_2_−C_4_H_8_]^+^		Levistilide A
84.62	C_19_H_18_O_3_	295.1335 (0.9)		280.1076[M+H−CH_3_]^+^, 277.1222[M+H−H_2_O]^+^, 262.0984[M+H−CH_3_−H_2_O]^+^, 249.1267[M+H−H_2_O−CO]^+^		Tanshinone IIA
87.2	C_19_H_22_O_2_	283.1695 (0.9)		265.1598[M+H−H_2_O]^+^, 223.1107[M+H−H_2_O−C_3_H_6_]^+^, 207.1137[M+H−H_2_O−C_3_H_6_−CH_3_]^+^		Miltirone
97.22	C_18_H_35_NO	282.2796 (1.5)		265.2540[M+H−NH_3_]^+^, 247.2422[M+H−H_2_O−NH_3_]^+^		(Z)-9-Octadecenamide
100.6	C_32_H_48_O_5_	513.3578 (0.7)		407.3295[M+H−CH_2_O_2_−C_2_H_4_O_2_]^+^, 173.1321[M+H−C_25_H_24_O]^+^		Acetyl-11-keto-β-boswellic acid

### Monthly Determination of Biochemical Parameters

The dynamic graph of changes of biochemical parameters in this study can be found at **Supplementary Material** (dynamic changes bubble chart in parameters with time).

#### Body Weight

Body weights were measured at monthly intervals to compare weight gain in the different groups. As shown in **Table 2**, high-fat diet feeding resulted in a faster increase in body weight than that observed in the normal group. After 4 months of feeding, animals in NXT group showed significantly lower body weight compared with the model animals (p<0.05 or p<0.01).

**Table 2:** Changes of body weight in different groups.

Time (months)	Normal group	Model group	NXT group
0	13.97 ± 0.80	15.83 ± 1.63	15.73 ± 0.93
1	17.43 ± 1.24	17.20 ± 0.72	15.43 ± 1.97
2	21.40 ± 1.25	25.03 ± 1.52	21.17 ± 2.47
3	26.10 ± 3.86	32.17 ± 1.53	25.73 ± 2.77
4	34.20 ± 1.73	44.87 ± 1.50^**^	33.73 ± 1.70^#^
5	40.63 ± 1.38	51.93 ± 1.76^**^	42.90 ± 2.10^##^
6	45.43 ± 2.10	57.13 ± 1.75^**^	51.67 ± 3.23^##^
7	50.83 ± 1.15	62.90 ± 1.57^**^	60.00 ± 2.76
8	54.53 ± 0.61	71.23 ± 1.99^**^	68.57 ± 0.80

Data represent mean ± SD. **P < 0.01 vs. normal group. ^#^P < 0.05 vs. model group. ^##^P < 0.01 vs. model group. n = 3.

#### Effect of NXT on Lipid Metabolism

**Figure 1:**
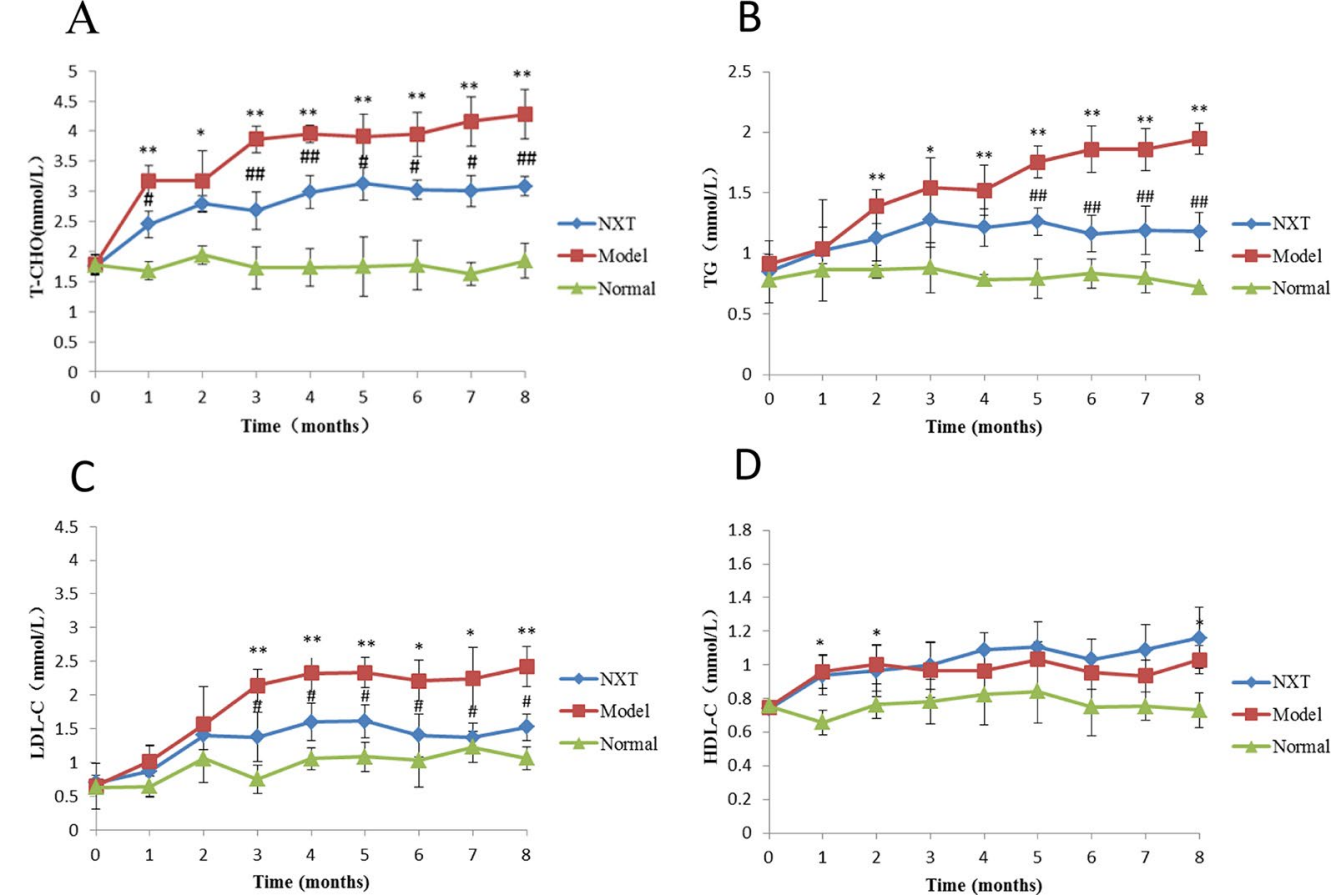
Changes in serum cholesterol (T-CHO) **(A)**, triglyceride (TG) **(B)**, low density lipoprotein (LDL-C), **(C)** and high-density lipoprotein (HDL-C) **(D)**. Data represent mean ± SD. *P < 0.05 vs. normal group. **P < 0.01 vs. normal group. ^#^P < 0.05 vs. model group. ^##^P < 0.01 vs. model group. n = 3.

The parameters of lipid metabolism were determined every month during the study. As shown in **Figure 1**, TC, TG, and LDL-C levels in the model group were increased significantly with time and were also significantly higher compared with normal group (p < 0.05 or p < 0.01) suggesting that the high-fat diet had induced hyperlipemia in minipigs. After drug administration, NXT significantly decreased the TC, TG, and LDL-C levels from the third month (p < 0.05 or p < 0.01).

#### Effect of NXT on Liver and Kidney Function

**Figure 2:**
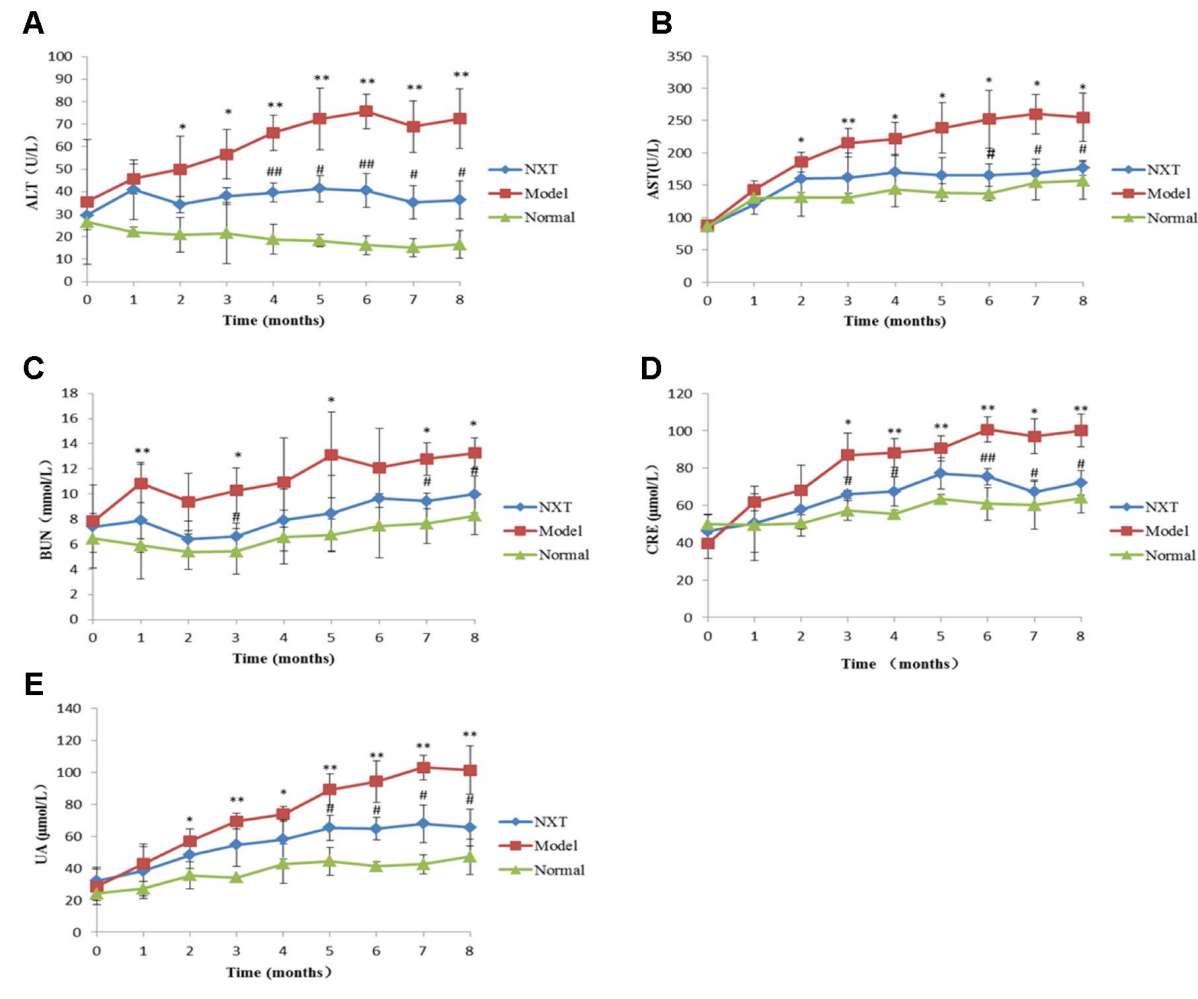
Changes in glutamic–oxalacetic transaminase (AST) **(A)**, glutamic–pyruvic transaminase (ALT) **(B)**, creatinine (Cr) **(C)**, urea nitrogen (BUN) **(D)**, uric acid (UA) **(E)**. Data represent mean ± SD. *P < 0.05 vs. normal group. **P < 0.01 vs. normal group. ^#^P < 0.05 vs. model group. ^##^P < 0.01 vs. model group. n = 3.

The liver and kidney function indexes of each group were examined once a month. As shown in **Figure 2**. The levels of ALT, AST, BUN, CRE, and UA in the model group increased significantly with time, and the levels of these indexes were significantly higher than those in the normal group (P < 0.05 or P < 0.01), indicating that minipigs in the model group exhibited liver and kidney dysfunction. From the fourth month, ALT, AST, BUN, CRE, and UA levels in NXT group were significantly lower than those in the model group (p < 0.05 or p < 0.01), indicating that NXT could play a role in protecting liver and kidney.

#### Effect of NXT on Oxidative Stress

**Figure 3:**
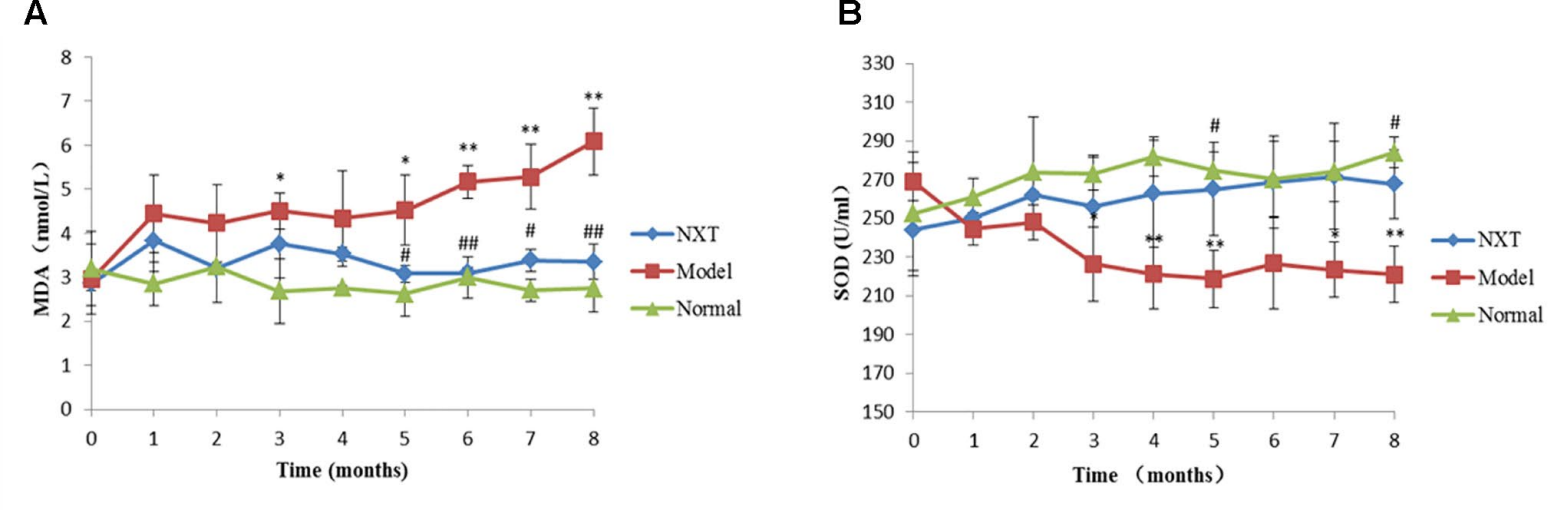
Changes in methane dicarboxylic aldehyde (MDA) **(A)**, superoxide dismutase (SOD) **(B)**. Data represent mean ± SD. *P < 0.05 *vs*. normal group. **P < 0.01 *vs*. normal group. ^#^P < 0.05 *vs*. model group. ^##^P < 0.01 *vs*. model group. n = 3.

SOD and MDA levels of each group were determined once a month. As shown in **Figure 3**, MDA level in the model group increased significantly with time, while the activity of SOD decreased. After 4 months high-fat diet feeding, MDA level in the model group was significantly higher than that in normal group, while SOD activity was significantly lower (P < 0.05 or P < 0.01), indicating that high-fat diet could induced oxidative stress disorders in minipigs. From the fifth month, NXT significantly decreased the MDA level and increased SOD activity (P < 0.05 or P < 0.01).

#### Effect of NXT on Inflammatory Responses

**Figure 4:**
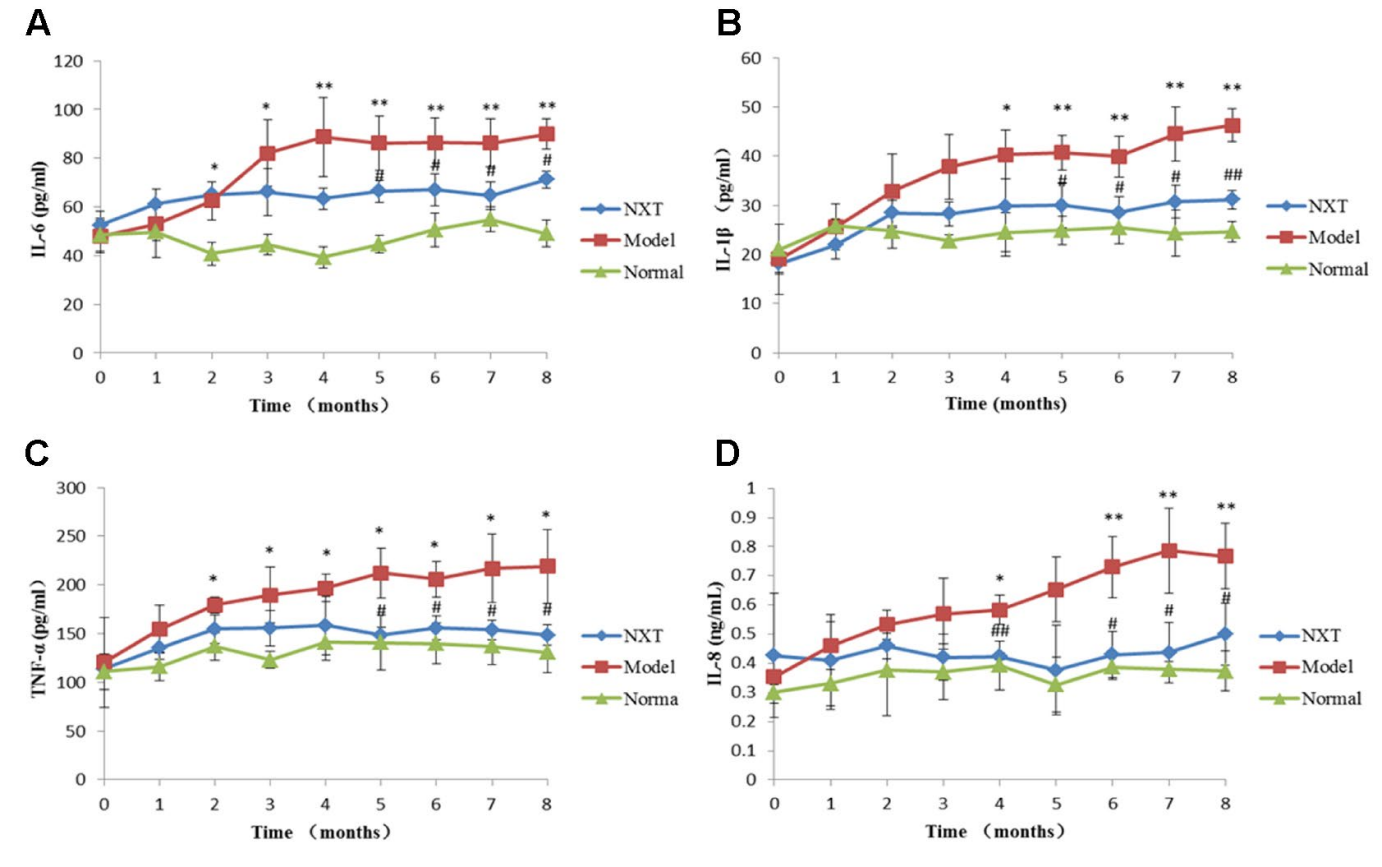
Changes in interleukin-6 (IL-6) **(A)**, interleukin-1β (IL-1β) **(B)**, tumor necrosis factor-α (TNF-α) **(C)**, and interleukin-8 (IL-8) **(D)**. Data represent mean ± SD. *P < 0.05 vs. normal group. **P < 0.01 vs. normal group. ^#^P < 0.05 vs. model group. ^##^P < 0.01 vs. model group. n = 3.

Serum inflammatory cytokines were determined every month. As shown in **Figure 4**, IL-6, IL-1β, TNF-α, and IL-8 levels in model group were increased with time, and the levels of these inflammatory cytokines were significantly higher than those in the normal group (P < 0.05 or P < 0.01), suggesting that high-fat diet caused obvious inflammation reaction in minipigs. From the fourth month, NXT significantly decreased the IL-6, IL-1β, TNF-α, and IL-8 levels (P < 0.05 or P < 0.01), indicating that NXT had the remarkable anti-inflammatory effect.

#### Effect of NXT on Myocardial Enzymes

**Figure 5:**
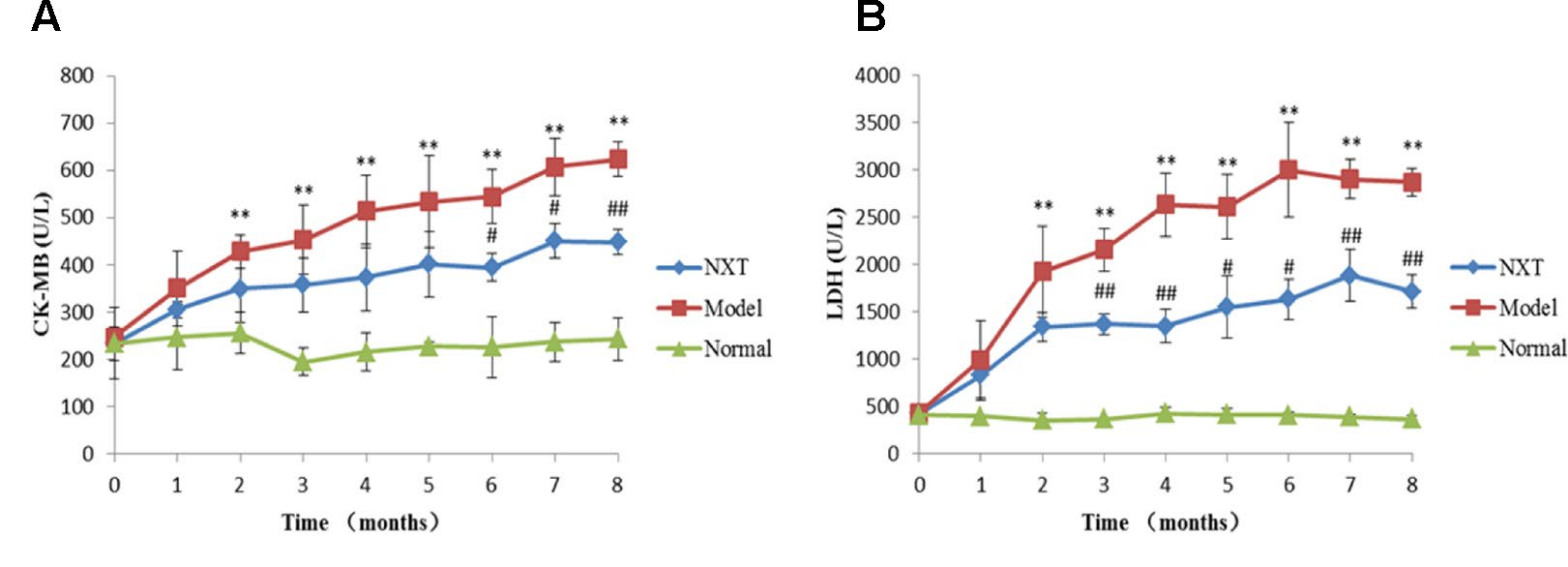
Changes in creatine kinase-MB **(A)** and lactic dehydrogenase **(B)**. Data represent mean ± SD. *P < 0.05 vs. normal group. **P < 0.01 vs. normal group. ^#^P < 0.05 vs. model group. ^##^P < 0.01 vs. model group. n = 3.

CK-MB and LDH levels of each group were determined once a month. As shown in **Figure 5**, the CK-MB and LDH levels in model group significantly increased over time, and were significantly higher than those in normal group (P < 0.05 or P < 0.01), suggesting the minipigs in model group exhibited myocardial injury. From the third month, CK-MB and LDH levels in NXT group were significantly lower than those in the model group (P < 0.05 or P < 0.01), indicating that the long-term administration of NXT could improve the myocardial injury.

### Pathological Observations of Tissues

**Figure 6:**
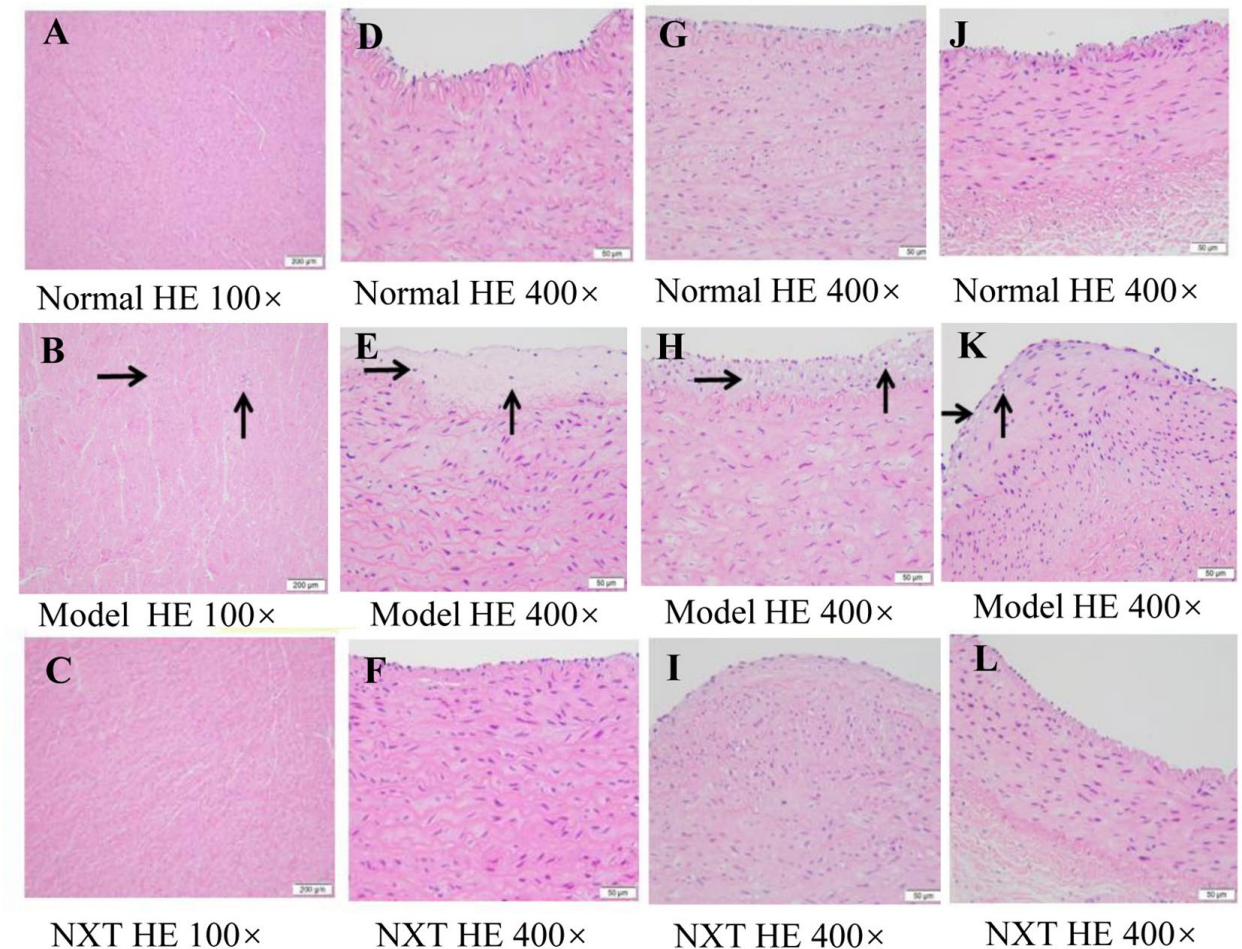
Pathological observations with hematoxylin-eosin (HE) staining on heart **(A**-**C)**, carotid artery **(D**-**F)**, abdominal aorta **(G**-**I)**, and coronary artery **(J**-**L)** of minipigs in each group.

HE staining results of cardiac tissue sections showed that the myocardial fibers of model group were loose. And fibrous tissue hyperplasia in the interstitium, a small amount of local myocardial fiber necrosis and inflammatory cell infiltration appeared in cardiac tissue in the model group (**Figure 6B**).The results of HE staining of arteries showed that subintimal lipid necrotic material deposition and a small amount of macrophage infiltration appeared in the carotid artery (**Figure 6E**), abdominal aorta (**Figure 6H**), and coronary artery (**Figure 6K**) in the model group. No abnormalities were observed in the normal group (**Figure 6A, D, G, J**) and NXT group (**Figure 6C, F, I, L**). Such pathological observation results indicated that NXT could inhibit the above vascular lesions caused by the long-term high-fat feeding.

### Effects of NXT on Intestinal Microecology

#### Diversity of Intestinal Microflora in the Minipigs After NXT Treatment

**Figure 7:**
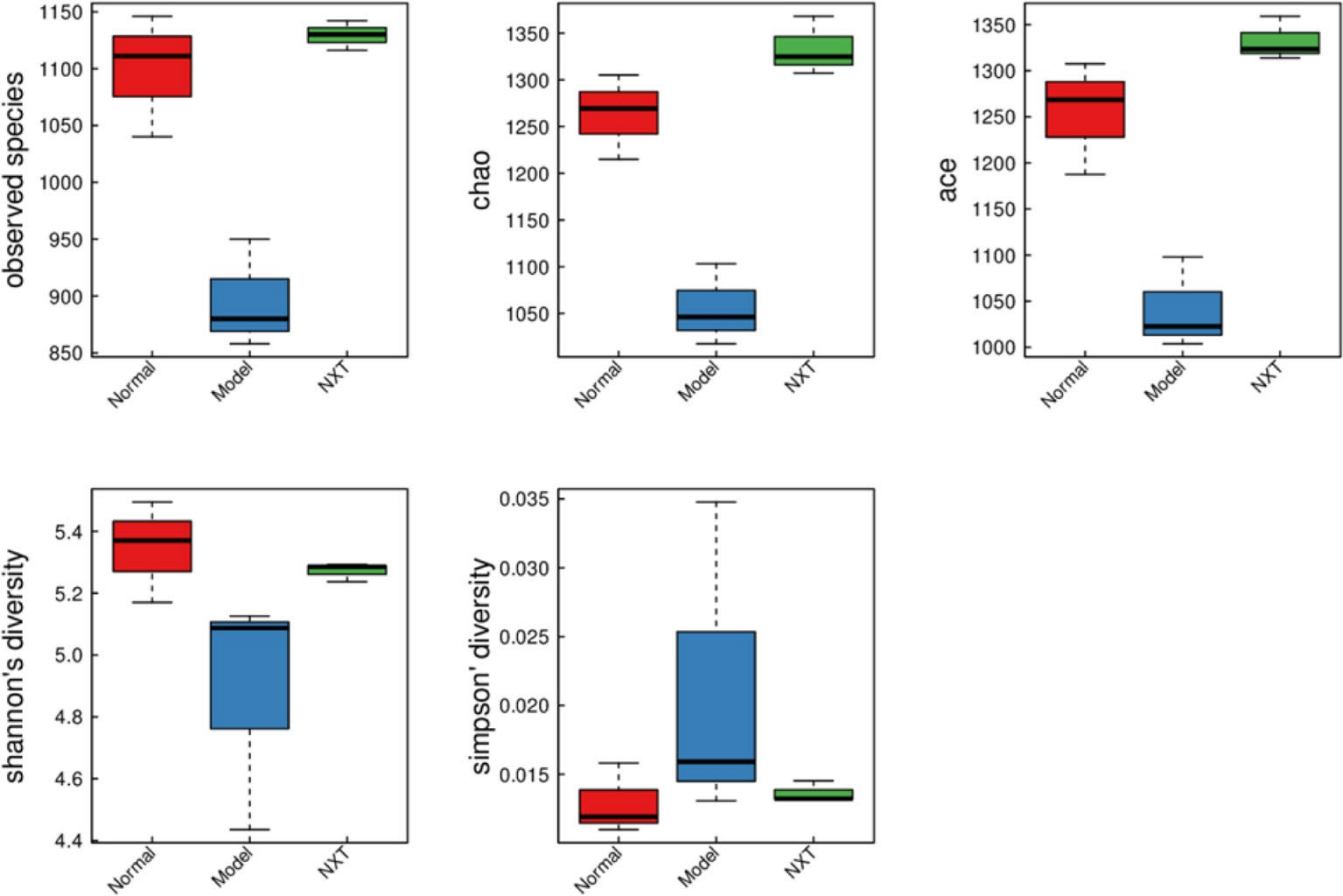
Box chart of alpha diversity between groups.

To investigate how NXT influenced the diversity of gut microbiota, the observed species index, Chao index, Ace index, Shannon index, and Simpson index were calculated to estimate the alpha diversity. The observed species index, Chao index, and Ace index are used to estimate the OUT number in microflora representing species richness. Shannon and Simpson indices are quantitative measures of bacterial diversity reflecting both species richness and evenness. The bigger the first four indexes are, as well as the smaller the Simpson index is, indicating that species in the sample is more abundant. As shown in **Figure 7**, the above indexes of each group suggested that the diversity of intestinal microbial of minipigs in the model group was significantly decreased. And the long-term administration of NXT could play a role in restoring the gut microbiota species diversity.

#### Beta Diversity Changes Due to the NXT Treatment

**Figure 8:**
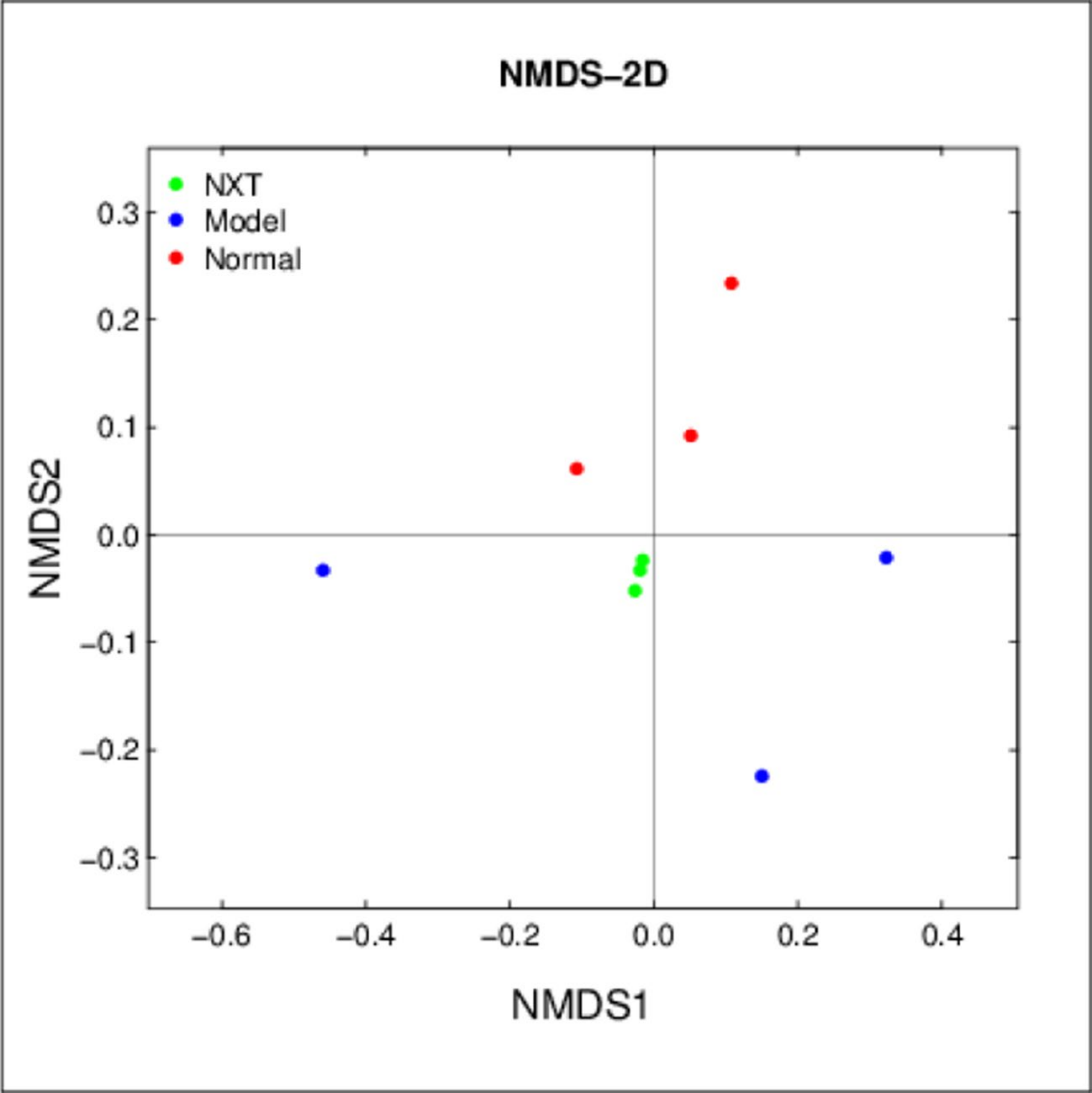
Beta diversity changes due to the NXT treatment. Samples of the same color belong to the same group (green, NXT group; blue, model group; red, normal group). The closer the samples are, indicating that the more similar the composition of the samples.

The three groups were clearly distinguished in NMDS (**Figure 8**), indicating that the composition of the gut microbiota differed between them. More importantly, the result showed that the distance differences among minipigs in the NXT group were very small, indicating the composition of gut microbiota in each minipig in NXT group was very similar. This finding suggested that the long-term administration of NXT had significant effect on regulating the microflora structure and the microflora structure in NXT group tend to be stable in our study.

#### Relative Abundances of Different Bacteria After NXT Treatment

**Figure 9:**
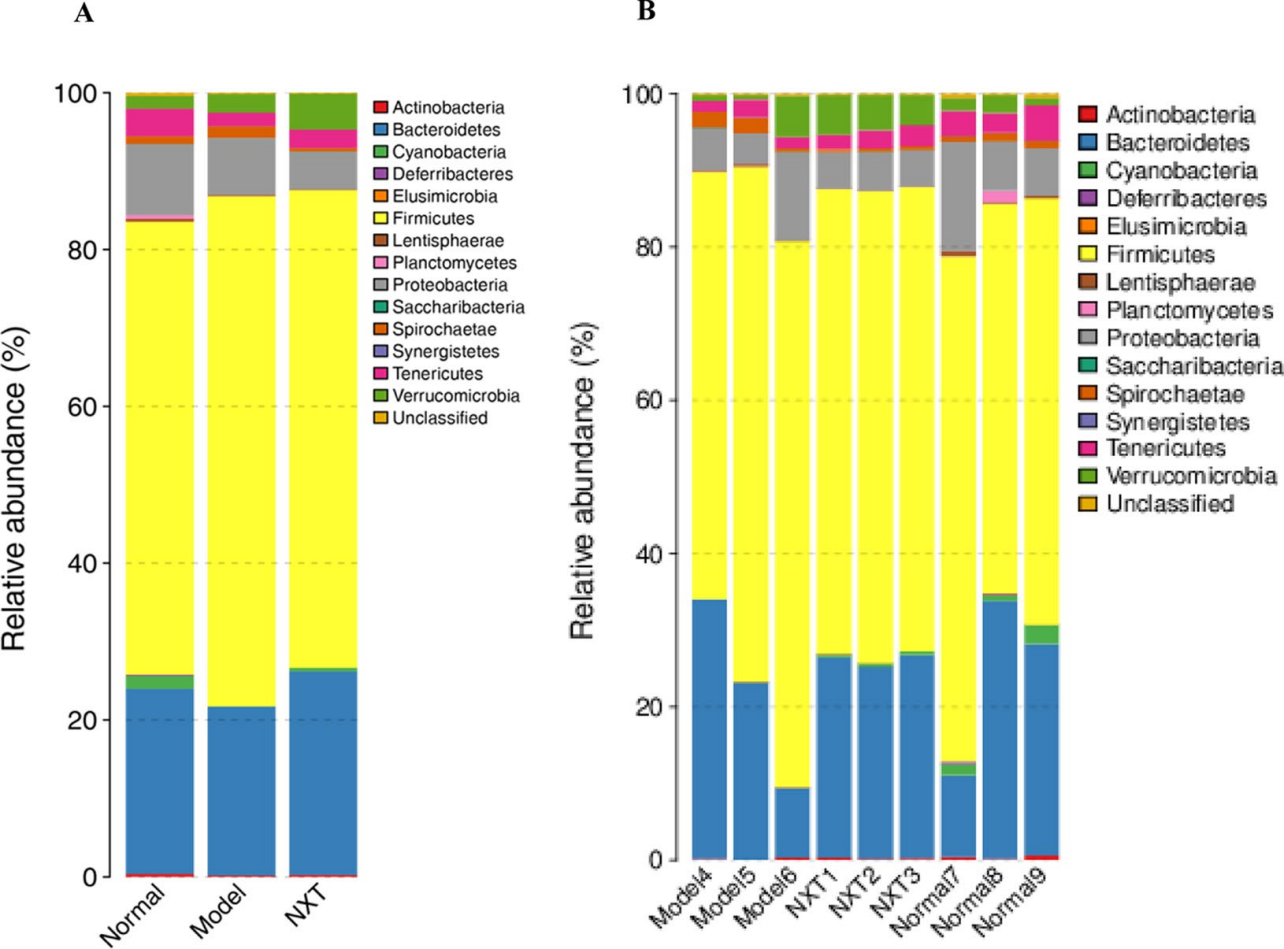
Relative abundances of different bacteria at phylum level of each group **(A)** and each animal **(B)**.

At the phylum level, the decreased abundance of *Bacteroidetes* and the increased abundance of *Firmicutes* were observed in the model group (**Figure 9A**), suggesting that the long-term high-fat diet feeding could cause the increase of the ratio of the *Firmicutes* to *Bacteroidetes* (F/B ratio) in relative abundance. The long-term administration of NXT increased the abundance of *Bacteroidetes* and decreased the abundance of *Firmicutes* in minipigs with high-fat diet feeding (**Figure 9A**). Our results showed that NXT treatment could revered the increase of the ratio of the *Firmicutes* to *Bacteroidetes*. Moreover, NXT treatment could reduce the relative abundance of *Proteobacteria* (**Figure 9A**). The result of relative abundances of different bacteria in each minipig (**Figure 9B**) showed that the microflora structure tended to be stable in NXT group, indicating that the long-term administration of NXT could steadily regulates the gut microbiota.

**Figure 10:**
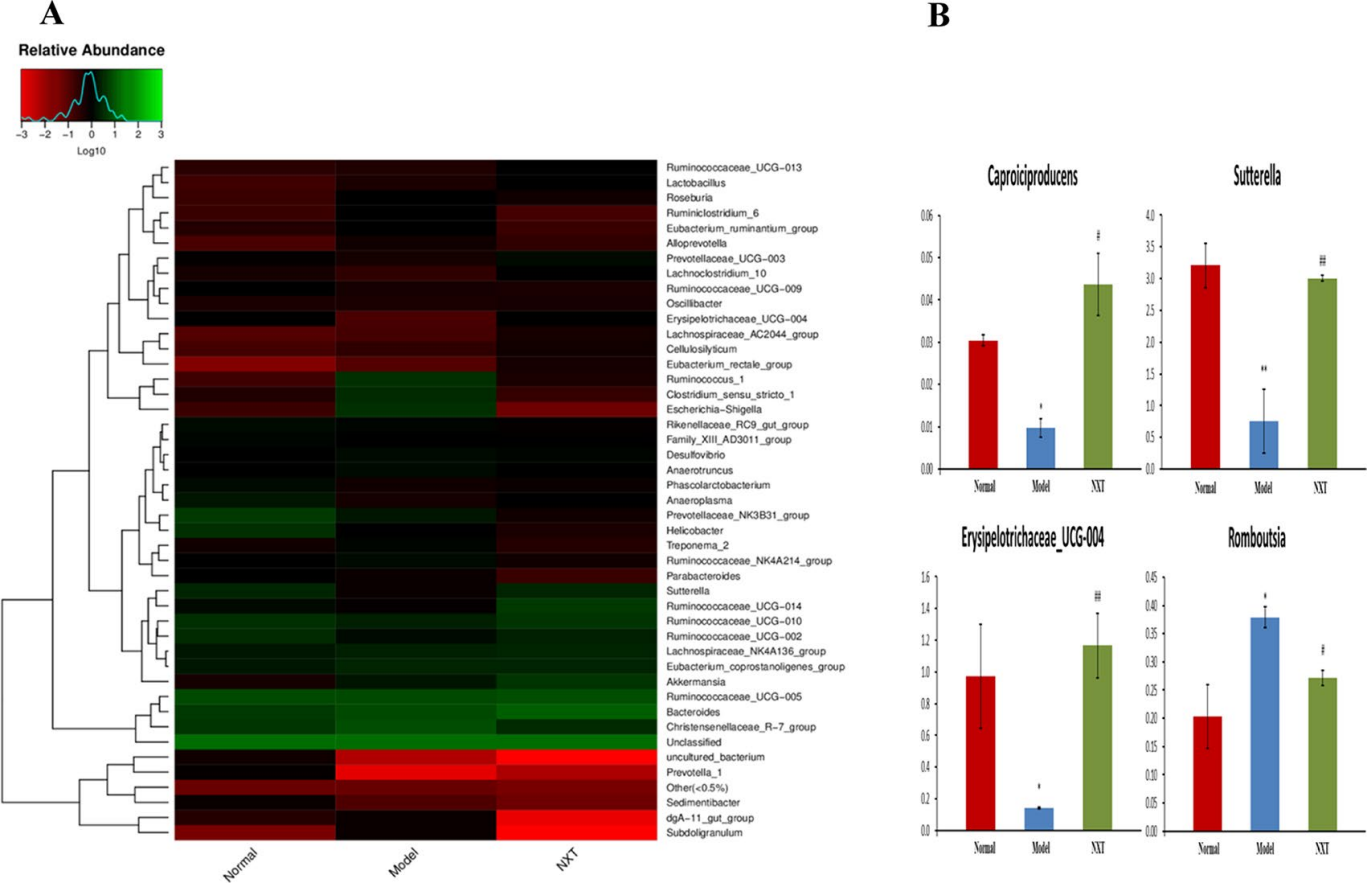
Effect of NXT on the recovery of dysbiosis of gut microbiota **(A)** and key bacteria **(B)** at genus level. For **(A)**, the higher red degree means the less the relative abundance, while the higher green degree means the more the relative abundance. For **(B)**, data represent mean ± SD. *P < 0.05 vs. normal group. ^#^P < 0.05 vs. model group.

Gut microbes species abundance distribution at the genus level were shown in **Figure 10A**. *Caproiciproducens*, *Sutterella*, and *erysipelotrichaceae_ucg-004* abundances were significantly reduced in model group compared to the normal group (P < 0.05, P < 0.01, P < 0.05), whereas the *Romboutsia* abundance was significantly elevated in the model group (P < 0.05). *Caproiciproducens*, *Sutterella*, and *erysipelotrichaceae_ucg-004* abundances were all significantly elevated by NXT treatment (P < 0.05, P < 0.01, P < 0.01), and *Romboutsia* abundance was significantly reduced by NXT treatment (P < 0.01, P < 0.05) (**Figure 10B**).

#### Correlation Between Bacterial Taxa and Pathological Measurements

**Figure 11:**
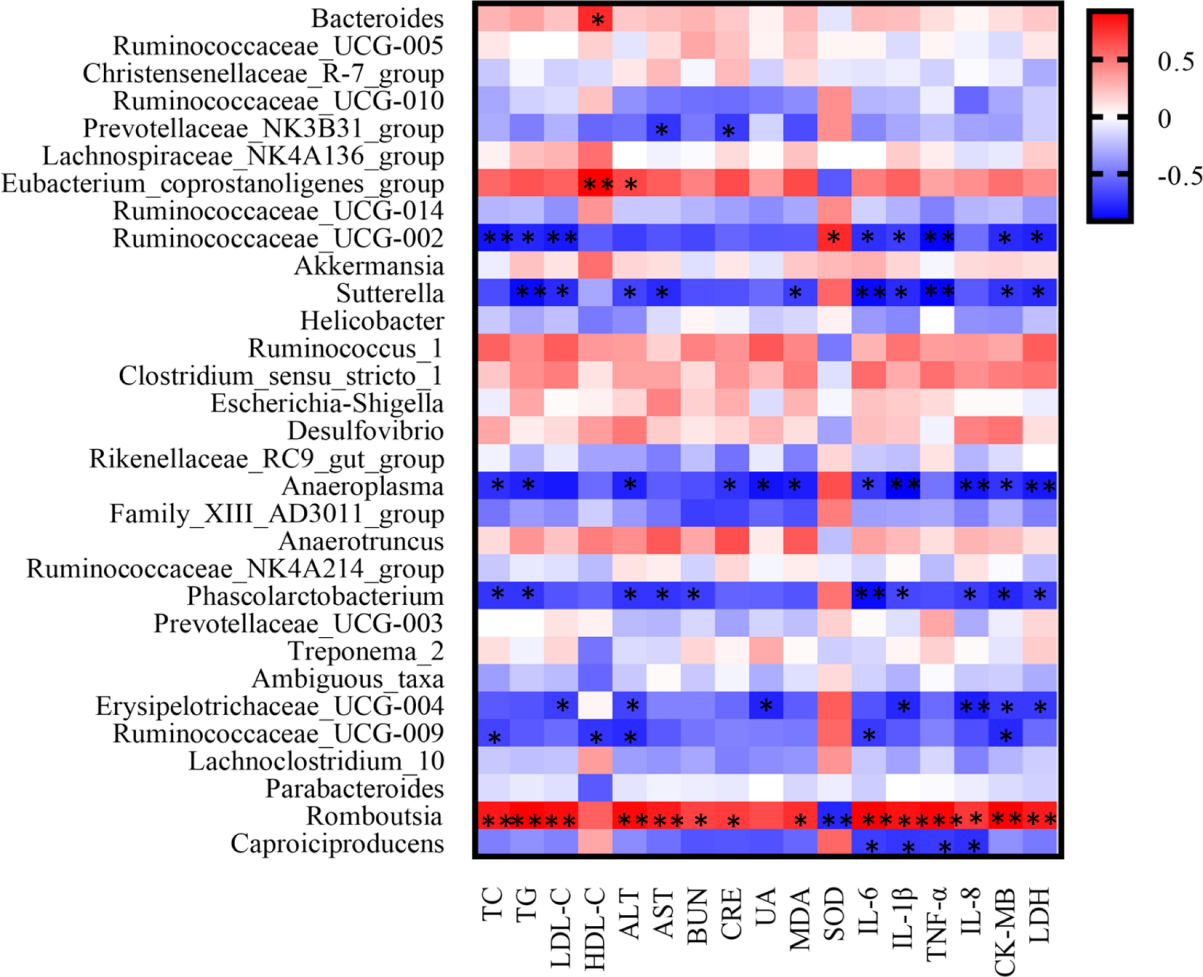
The correlation heatmap of identifying associations between the gut microbiota structure (genus level) and the pathological indexes. The red color means positive correlation while the blue color shows a negative correlation. The deeper color means the greater correlation (p < 0.05*, p < 0.01**).

**Figure 12:**
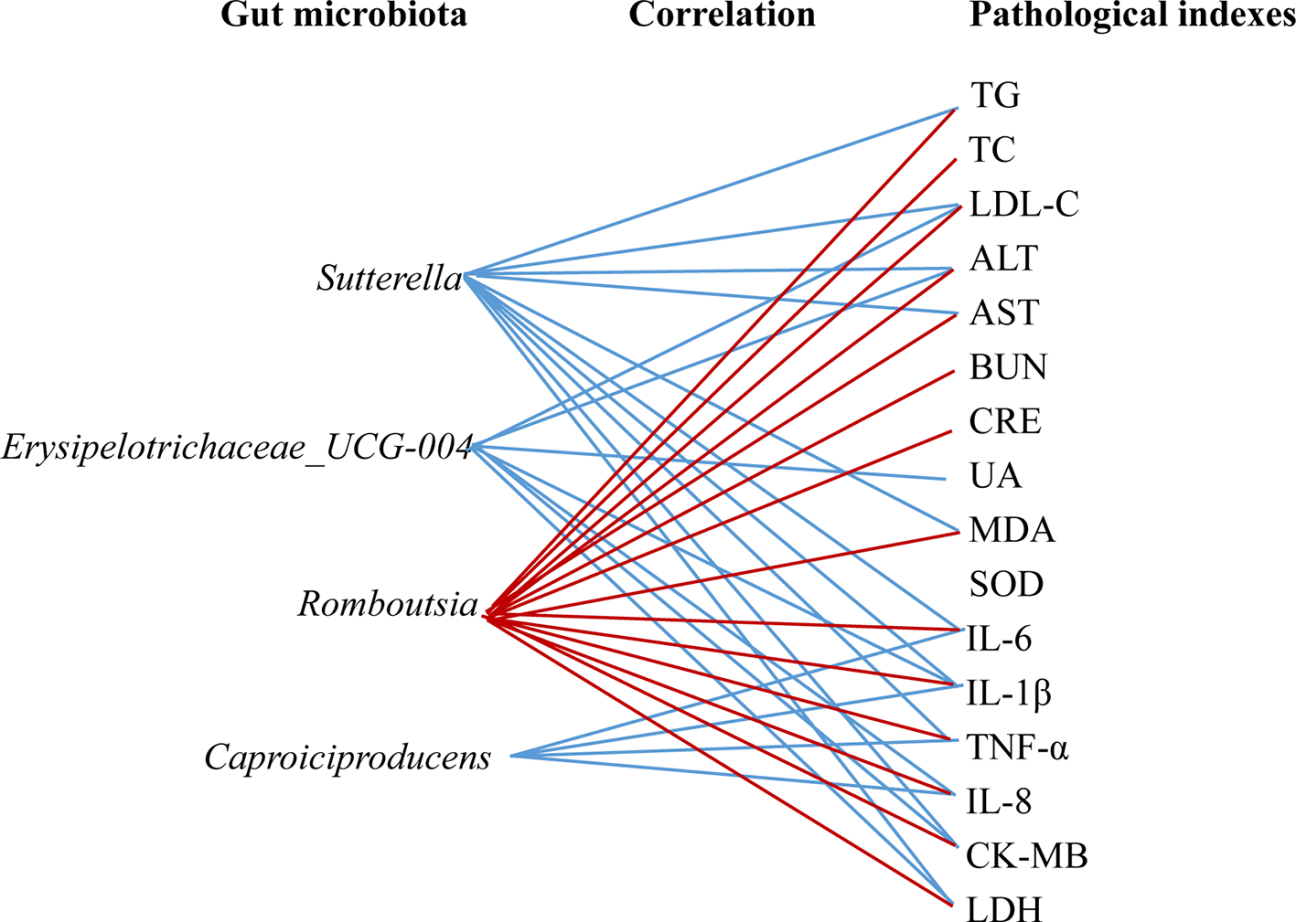
Associations between the gut microbiota (NXT significantly affect) and the pathological indexes. Associations with the pathological indexes are shown for each genus microbiota with the direction of correlation indicated by red (positive) or blue (negative) lines.

As shown in **Figure 11**, the covariation between pathological indexes and 16SrDNA data are presented in the form of a heatmap diagram. Most of the gut microbiota had the correlation with multiple pathological indexes. For the gut microbiota, NXT signiﬁcantly affect; *Sutterella* had a negative correlation with TG, LDL-C, ALT, AST, MDA, IL-6, IL-1β, TNF-α, CK-MB, and LDH. *Erysipelotrichaceae_UCG-004* had a negative correlation with LDL-C, ALT, UA, IL-1β, IL-8, CK-MB, and LDH. *Caproiciproducens* had a negative correlation with IL-6, IL-1β, TNF-α, and IL-8. While *Romboutsia* had a positive correlation with TC, TG, LDL-C, ALT, AST, BUN, CRE, MDA, IL-6, IL-1β, TNF-α, IL-8, CK-MB, and LDH (**Figure 12**).

## Discussion

In the present study, UFLC-Q-TOF-MS/MS was used to identify constituents in NXT. A total of 59 compounds were identified, and most of these active components were reported to have potential anti-CVD effects. In addition, we observed and evaluated the effect of long-term administration of NXT on minipigs with high-fat diet feeding. And we found that, in the course of time, NXT treatment effectively improved the blood lipid metabolism, inhibited vascular inflammation, enhanced antioxidant capacity, and alleviated myocardial injury. Moreover, NXT treatment could restore the imbalance of gut microbiota caused by the high-fat diet. And, we found some key bacterium of *Caproiciproducens* (enhanced), Sutterella (enhanced), *Erysipelotrichaceae* (enhanced), and *Romboutsia* (decreased) were closely involved in NXT’s effects in our study.

Serum TC refers to the sum of all lipoprotein cholesterol in the blood; the increased content of TC has become the independent risk factor for coronary heart disease (Cui et al., 2007). Higher TG level is closely associated with the risk of atherosclerotic cardiovascular disease (Bittner, 2001). LDL contributes to the deposition of cholesterol in blood vessel wall, and the increased content of LDL could lead to cardiovascular disease (Jeppesen et al., 2006). In the present study, in model animals with high-fat diet feeding, the TC, TG, and LDL-C levels increased significantly with breeding time, and from 4th month, the contents were significantly higher than those of normal group. Our results showed that high-fat feeding for 4 months could cause the lipid metabolic abnormalities in minipigs. And, long-term administration of NXT significantly inhibited the increase of TC, TG, and LDL-C thus improved hyperlipemia effectively.

IL-1β is an important mediator in the inflammatory response, which can be secreted and synthesized by a variety of cells (Prantner et al., 2009). It can aggravate the tissue damage mediated by cellular immunity and humoral immunity, promote the expression of endothelial leukocyte adhesion molecule, and stimulate the migration of inflammatory cells into the lesion site. IL-6 is an inflammatory factor that plays an important role in vascular injury and acute myocardial ischemia (Ridker et al., 2000). The increase of IL-6 caused by the injury of vascular endothelium could induce a large number of corresponding antibodies to form immune complexes and result in thrombosis. IL-8 is a cytokine involved in immune regulation and inflammatory response, which plays an important role in the aggregation and activation of neutrophils in various inflammatory processes, and its level is associated with the stability of atherosclerotic plaques (Frostegård et al., 1999). TNF-α is a polypeptide produced by the activated monocytes and lymphocytes, which can promote blood coagulation and inhibit fibrinolysis, and it is closely related to the occurrence and development of atherosclerosis (Leboeuf and Schreyer, 1998). In our study, the serum IL-6, IL-8, IL-1, and TNF-α levels of minipigs in model group increased continuously with the passage of high-fat feeding time, suggesting that vascular endothelium of minipigs suffered from damage during high-fat diet feeding, thus leading to the up-regulation of inflammatory factors. Long-term administration of NXT inhibited the gradual increase of the above inflammatory factors, indicating that NXT could protect the vascular endothelium and had remarkable anti-inflammatory effect.

The activity of ALT and AST would sensitively increase when the liver cells are injured or necrotic. Serum ALT and AST levels were significantly increased in the model group, indicating that prolonged high-fat diet feeding caused the liver dysfunction in minipigs. The long-term administration of NXT could reduce the levels of ALT and AST in serum, indicating that NXT had a certain protective effect on liver. In addition, the significant increased levels of Cr, BUN, and UA in minipigs in the model group suggested the dysfunction of glomerular filtration and renal tubular secretion. NXT treatment inhibited the abnormal elevated levels of Cr, BUN, and UA, which indicated that NXT had the protective effect on the kidney. The increase of reactive oxygen species (ROS) and the damage of endogenous antioxidants would cause the imbalance of redox state and inflammation, which further aggravate the damage to tissues and organs. A significant reduction in the antioxidant capacity of the minipigs with long-term high-fat diet was determined as the increase of MDA content and the decrease of SOD activity. NXT inhibited both the rise of MDA content and the decrease of SOD activity, suggesting that NXT could inhibit the aggravation of oxidative stress disorder. CK-MB is released into the bloodstream when myocardial cells are injured, so its level can reflect the degree of injury to myocardial cells (Adams et al., 1994). LDH is commonly used to diagnose the occurrence of myocarditis in clinic (Snodgrass et al., 1959). In the model animals, the levels of CK-MB and LDH were significantly increased, and hyperplasia of cardiac fibrosis, myocardial fibrosis necrosis, and inflammatory cell infiltration were observed in the heart issue (**Figure 6B**). The NXT treatment could effectively decrease the level of CK-MB and LDH and thus prevent the relevant lesion in the myocardial issue (**Figure 6C**).

In recent years, the increasing number of studies have revealed a close relationship between gut microbiota and blood lipid metabolism (Fava et al., 2006; Velagapudi et al., 2010). Some studies found that *Bifidobacterium*, *Lactobacillus*, *Enterococcus*, and etc. could produce conjugated bile acid hydrolytic enzyme. Such enzyme can transform conjugated bile acid into free bile acid; thereby, the enterohepatic circulation of bile acid will be affected, and the synthesis of bile acid in the liver will be promoted, which makes the cholesterol level in the blood reduce (Hlivak et al., 2005; Mishra and Prasad, 2005; Wang et al., 2009). On the other hand, the intestinal microecology will be changed when hyperlipidemia developed. The changes in intestinal microecology will affect the metabolism and reproduction of some normal gut microbiota and thus make their quantity decreased significantly, such as *Bifidobacteria*, *Lactobacillus*, *Enterococcus*, and etc., whereas, the relative abundance of *Enterobacteriaceae* increases, thereby the dysbacteriosis developed. The long-term high-fat diet feeding can cause the prolong and continuous changes in intestinal microecology (Ley et al., 2006). In the present study, minipigs were fed with high-fat diet up to 8 months, and blood lipid metabolism disorder developed. Alpha diversity results showed that the diversity of gut microbiota of minipigs in the model group was significantly decreased (**Figure 7**). Our result was consistent with the finding of De WIT N et al., which demonstrated that high-fat diet could reduce the diversity of gut microbiota (De et al., 2012). And, the long-term administration of NXT could restore the diversity of gut microbiota in our study, suggesting that NXT might improve the blood lipid metabolism disorder by ameliorating the dysbacteriosis.

Various kinds of diseases have been shown to be associated with the gut microbiota recently. It has been reported that an elevated ratio of *Firmicutes* to *Bacteroidetes* was significantly associated with cardiovascular disease, obesity, and diabetes (Mariat et al., 2009; Everard and Cani, 2013; Chang et al., 2017). In this study, the high-fat diet for up to 8 months could decrease the abundance of *Bacteroidetes*, increase the abundance of *Firmicutes*, and elevate ratio of *Firmicutes* to *Bacteroidetes* in minipigs in the model group (**Figure 10A**). Our results were in accordance with the previous studies. Ley R E *et al*. found that, compared with lean people, the abundance of *Bacteroidetes* in the obese people was lower, and the abundance of *Firmicutes* was higher, and the proportion of *Firmicutes*/*Bacteroidetes* was significantly increased (Ley et al., 2006). Guo et al. compared the abundance of *Bacteroidetes* and *Firmicutes* in obese and thin minipigs and found that the abundance of *Bacteroidetes* was significantly reduced in obese minipigs (Guo et al., 2010). And, in the present study, our results showed that the long-term administration of NXT could increase the abundance of *Bacteroidetes* and reduce the abundance of *Firmicutes* thus revered the increase of the ratio of the *Firmicutes* to *Bacteroidetes* in relative abundance caused by the high-fat diet.

More importantly, at the phylum level, the result of relative abundances of different bacteria in each minipig in NXT group (**Figure 10B**) showed that the microflora structure tended to be stable after 8 months of treatment. And the result of Beta diversity also showed that the composition of gut microbiota in each minipig in NXT group was very similar (**Figure 8**). In other words, the long-term NXT treatment could improve the composition of gut microbiota stably. This exciting finding suggested that the long-term administration of NXT was capable of reversing varied compositional abnormalities (dysbiosis) of gut microbiota associated with blood lipid metabolism disorders by steadily modulating the composition of gut microbiota instead of altering the composition of intestinal microbial community simply.

At the genus level, we found that NXT treatment significantly increased *Caproiciproducens*, *Sutterella*, and *Erysipelotrichaceae_UCG-004* abundance while reduced *Romboutsia* abundance (**Figure 10B**). *Caproiciproducens* is an acid-producing bacterium that can use galactose as the carbon source. The end products of its anaerobic fermentation are hydrogen, carbon dioxide, ethanol, acetic acid, butyric acid, and caproic acid (Liu et al., 2017). Caproic acid has many effects, such as inhibiting pathogenic bacteria, improving gut microbiota, promoting animal growth, and enhancing animal immunity (Van et al., 2004). This result indicated that NXT might improve the body’s immune by increasing *Caproiciproducens* abundance to produce more caproic acid. And, by this way, NXT could inhibit pathogenic bacteria and improve the gut microbiota. Moreover, the long-term high-fat diet feeding could result in a significant higher abundance of *Romboutsia* in minipigs (**Figure 10B**). This result was consistent with the experimental results of Liu Han *et al*., which demonstrated that a high-sugar and high-fat diet could increase the relative abundance of *Romboutsia* (Han et al., 2018). And NXT treatment could reduce the relative abundance of *Romboutsia* in our study. The result of correlation analysis between gut microbiota and pathological indexes showed that *Romboutsia* had a positive correlation with blood lipid profiles, hepatic and renal functions, inflammation cytokine, and myocardial enzyme. While *Caproiciproducens*, *Sutterella*, and *Erysipelotrichaceae_UCG-004* had a negative correlation with blood lipid profiles, inflammation cytokine, and myocardial enzyme (**Figures 11**, **12**). These results suggested that the modulation of gut microbiota might be one mechanism by which NXT prevent and treat cardiovascular diseases, which warrants a further investigation on the specific involvement of gut microbiota in NXT’s cardioprotective effects.

## Conclusions

The present study demonstrated that NXT was effective in reducing blood lipids, inhibiting vascular inflammation, enhancing antioxidant capacity, and alleviating myocardial injury, without damages on liver and kidney particularly. On the other hand, NXT played an important role in recovering the imbalance of intestinal microecology. These results indicated that NXT had integrated regulating effect on the prevention and treatment in cardiovascular diseases partly through ameliorating high-fat diet–induced metabolic disorders and partly through stably improving gut microbiota. The findings of this study would provide a further reference for the clinical application of NXT.

## Data Availability Statement

The datasets generated for this study can be found in Genebank using the accession number KDAE00000000.

## Ethics Statement

The animal study was reviewed and approved by Animal Ethics Committee of the School of Life Sciences in Sun Yat-sen University.

## Author Contributions

W-JZ, HL, H-LY and W-WS conceived and designed the experiments; Q-WL, H-YR, Z-HY and XZ conducted experiments; W-JZ, T-BC and HL analyzed the data; W-JZ, HL and P-BL wrote the manuscript.

## Funding

This work was supported by the grants of Secondary Development Projects of Traditional Chinese Medicine (2017-No.19), Project funded by China Postdoctoral Science Foundation under grant number (2018T110857, 2018M643036) and Guangzhou Major Special Projects of People’s Livelihood (201704020119, 201803010082). The funders had no role in the study design, data collection and analysis, decision to publish, or preparation of the manuscript.

## Conflict of Interest

The authors declare that the research was conducted in the absence of any commercial or financial relationships that could be construed as a potential conflict of interest.

## Supplementary Material

Figure S1 The identity of chemical constituents of NXT in different batches (A: positive ionization mode, B: negative ionization mode)
