# Supplementary figures and images for: Naoxintong Capsule Inhibits the Development of Cardiovascular Pathological Changes in Bama Minipig Through Improving Gut Microbiota
Source: Front Pharmacol. 2019 Oct 3;10:1128. doi: 10.3389/fphar.2019.01128 (PMC6785636; doi:10.3389/fphar.2019.01128)

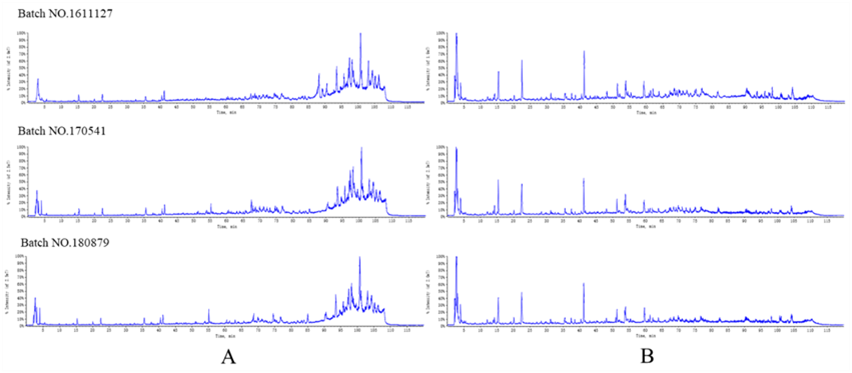

Supplement: Figure S1 — The identity of chemical constituents of NXT in different batches (A: positive ionization mode, B: negative ionization mode) [file Image_1.tif]
